# Dissemination of Genetic Acquisition/Loss Provides a Variety of Quorum Sensing Regulatory Properties in *Pseudoalteromonas*

**DOI:** 10.3390/ijms19113636

**Published:** 2018-11-18

**Authors:** Zhiliang Yu, Yajuan Ding, Jianhua Yin, Dongliang Yu, Jiadi Zhang, Mengting Zhang, Mengdan Ding, Weihong Zhong, Juanping Qiu, Jun Li

**Affiliations:** College of Biotechnology and Bioengineering, Zhejiang University of Technology, Hangzhou 310014, China; 2111505006@zjut.edu.cn (Y.D.); jianhuay@zjut.edu.cn (J.Y.); 2111605007@zjut.edu.cn (D.Y.); 2111705002@zjut.edu.cn (J.Z.); 2111705006@zjut.edu.cn (M.Z.); 2111705005@zjut.edu.cn (M.D.); whzhong@zjut.edu.cn (W.Z.); qiujping@zjut.edu.cn (J.Q.)

**Keywords:** quorum sensing, *Pseudoalteromonas*, regulatory architecture, dissemination, horizontal transfer

## Abstract

Quorum sensing (QS) enables single-celled bacteria to communicate with chemical signals in order to synchronize group-level bacterial behavior. *Pseudoalteromonas* are marine bacteria found in versatile environments, of which QS regulation for their habitat adaptation is extremely fragmentary. To distinguish genes required for QS regulation in *Pseudoalteromona*s, comparative genomics was deployed to define the pan-genomics for twelve isolates and previously-sequenced genomes, of which acyl-homoserine lactone (AHL)-based QS traits were characterized. Additionally, transposon mutagenesis was used to identify the essential QS regulatory genes in the selected *Pseudoalteromonas* isolate. A remarkable feature showed that AHL-based colorization intensity of biosensors induced by *Pseudoalteromonas* most likely correlates with QS regulators genetic heterogeneity within the genus. This is supported by the relative expression levels of two of the main QS regulatory genes (*luxO* and *rpoN*) analyzed in representative *Pseudoalteromonas* isolates. Notably, comprehensive QS regulatory schema and the working model proposed in *Pseudoalteromona*s seem to phylogenetically include the network architectures derived from *Escherichia coli*, *Pseudomonas,* and *Vibrio*. Several associated genes were mapped by transposon mutagenesis. Among them, a right origin-binding protein-encoding gene (*robp*) was functionally identified as a positive QS regulatory gene. This gene lies on a genomic instable region and exists in the aforementioned bioinformatically recruited QS regulatory schema. The obtained data emphasize that the distinctly- and hierarchically-organized mechanisms probably target QS association in *Pseudoalteromona*s dynamic genomes, thus leading to bacterial ability to accommodate their adaption fitness and survival advantages.

## 1. Introduction

Newly characterized in 1995, the *Pseudoalteromonas* genus is ubiquitously present in marine environments, and is able to synthesize broad bioactive chemical molecules [1]. They are greatly versatile bacteria existing in diverse life habitats, as well as on animal and plant tissues, such as *Pseudoallteromonas tunicate* isolated from the sea lions, *Pseudoalteromonas citrea* isolated from sponges, and *Pseudoalteromonas nigrifaciens* isolated from mussels [2,3,4,5]. Broad life habitats suggest their versatile evolution in adaptation patterns. *Pseudoalteromonas* is a genus of ubiquitous marine bacteria typically recognized as model organism to explore the adaptation/fitness mechanisms under extreme conditions [6].

In fact, the genomes of several *Pseudoalteromonas* spp. have been sequenced, evidencing their genetic heterogeneity accounting for different adaptation capabilities [6,7]. Genomic analysis uncovered the genetic basis of *Pseudoalteromonas flavipulchra* JG1 in marine environment [1]. The potential fitness of *Pseudoalteromonas haloplanktis* TAC125 to cold environments was elucidated by genome sequencing [8]. The comparative genomics analysis on *Pseudoalteromonas tunicata* D2 has revealed the adaptation mechanism of its epiphytic life on algae surface [9]. Whole genome sequencing, together with comparative genomics analysis within *Pseudoalteromonas* sp. SM9913 and *P*. *haloplanktis* TAC125, have illustrated the adaption of *Pseudoalteromonas* to marine sediment environments as well [10].

Diverse studies have proven that bacteria use quorum sensing (QS) to coordinate the population behaviors through cellular information exchange for adaption/fitness to complex and versatile environments [11]. QS is the unique process by which bacteria produce, release, and detect specific chemical signal molecules, and successively alter the expression of specific genes to maintain population replication/fitness for survival benefit [12]. By QS-related strategy, *Pseudomonas aeruginosa* alters adaptation abilities in different external environments [13,14]. QS is very important for surface- and biofilm-living bacteria that often reach high densities of microbiota [15]. QS is involved not only in the production of virulence factor and biofilm formation, but also bioluminescence, sporulation, motility, and antibiotic production [11,12,13,14,15,16].

First discovered in marine *Vibrio fischeri*, QS has now been determined in both Gram-positive and Gram-negative bacteria [16]. However, very few reports have dealt with QS in *Pseudoalteromonas*. It was found that QS is probably involved in the violacein production in *Pseudoalteromonas* [17,18]. The putative promoter regions of gene clusters induced by population-dependent signaling molecules were mapped in violacein-producing *Pseudoalteromonas* sp. 520P1 [19]. QS-related swimming motility and swarming motility of *Pseudoalteromonas* sp. SM9913 provided a mechanistic insight into its multifaceted lifestyle in deep-sea sediment [20]. Biofilm formation in *Pseudoalteromonas ulvae* TC14 was found to be influenced by heterogenous QS regulatory molecules [21].

Previous studies on QS in *Pseudoalteromonas* were merely focused on the identification of signal molecules and their cognate genes, together with QS regulation on bacterial physiology [22]. Herein, we explored the correlation between environmental adaptation potential and QS architecture from the 12 sequenced *Pseudoalteromonas* isolates. We also reported transposon (mini-Tn*10*) mutagenesis to identify QS-related regulators in *Pseudoalteromonas*. Several QS regulatory genes were successfully mapped, and right origin-binding protein-encoding gene (*robp*) was demonstrated to serve as a positive QS regulatory gene that localizes on a genomic instable region. The obtained results provide a better understanding of QS regulation in *Pseudoalteromonas*, highlighting the importance of horizontal gene transfer in their adaptation capacities.

## 2. Results

### 2.1. Characterization of Quorum Sensing Related Colorization Derived from Pseudoalteromonas Isolates

Based on 16S rRNA gene sequencing, the taxonomy of total bacteria isolated from the Mangrove forest sediments in Ximen Island (28°20′33′′N, 121°10′28′′E) of Zhejiang Province was determined. 16S rRNA sequence comparison indicated that the 12 isolates belong to *Pseudoalteromonas* (Table 1). AHLs-related colorization was then tested by cross-feeding bioassay using *A*. *tumefaciens* A136 as a biosensor [23] on a LB-2216E plate. The results in Table 1 showed that 11 out of 12 isolates result in the colorization of A136, and different isolates exhibit distinguished colorization intensities, indicating diverse capabilities of AHLs production. T1lg65 yields the strongest colorization, whereas T1lg88 does not result in any visualizable colorization. Based on these scenarios, we speculate that genetic patterns and genomic discrepancy among different *Pseudoalteromonas* isolates probably influence their traits in producing QS signals. Data in Supplementary Figure S1 indicated that there is no direct correlation between the intensity of QS signals and biofilm formation or motility within these 12 isolates.

### 2.2. Genomic Quality Control and Diversified Sequence Features

The genomes of the 12 *Pseudoalteromonas* isolates were then sequenced and analyzed. It was found that genomic size, protein-coding gene numbers, and G+C contents significantly vary in the 12 *Pseudoalteromonas* isolates (Table 2), consistent with a previous report [24] reflecting genomic heterogeneity.

The identified regulators listed in Supplementary Table S1 further show that the 12 *Pseudoalteromonas* isolates have significant genomic heterogeneity. Given that T1lg65 facilitates the strongest colorization of A136, T1lg48 moderate colorization, T1lg24 weak colorization, and T1lg88 no colorization (Table 1), the comparative genomics of these *Pseudoalteromonas* isolates was performed using *Pseudoalteromonas* sp. SM9913 as control. The correlation between genomic heterogeneity and QS trait was accordingly exploited. The Venn diagram in Supplementary Figure S2 indicated that the non-conserved and specific genes in *Pseudoalteromonas* isolates with different QS traits are significantly different. For example, 1400 genes in T1lg65 are unique, whereas 412 genes in T1lg88 are specific.

Additionally, regulatory protein family classification was also performed to further investigate genetic patterns and QS-associated genes. The COG categories within T1lg65 and T1lg88 with opposite QS traits were determined by using SM9913 as control (Supplementary Figure S3). T1lg65 and T1lg88 are different in genomic size. Additionally, the orthologous proteins pertaining to COG ‘T’ and ‘J’, related to signal transduction mechanisms, and translation, ribosomal structure, and biogenesis, respectively, are obviously different in amount. The number of non-conserved sequences of ‘T’ in T1lg65 is higher than that in both T1lg88 and SM9913. Meanwhile, the number of conserved sequences of ‘J’ is high in all three representatives. The number of non-conserved sequences of ‘J’ in T1lg65 is higher than that in both T1lg88 and SM9913. All these data suggest a correlation between genomic heterogeneity and QS trait.

### 2.3. Dissemination of Regulatory Factors within Specific Genetic Regions in Pseudoalteromonas

**Figure 1:**
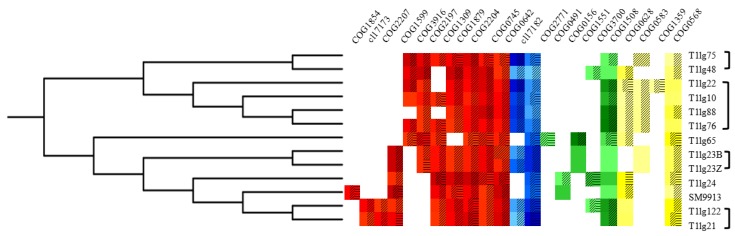
Heatmap schema of quorum sensing regulator dissemination in selected *Pseudoalteromonas* genomes. The left panel of the heatmap is the evolutionary tree. The middle of the heatmap denotes the distribution of QS regulatory protein families in thirteen *Pseudoalteromonas* isolates. The top of the heatmap corresponds to the protein family IDs. Red: positive regulation; blue: both positive and negative regulation; green: negative regulation; yellow: unknown regulation direction. The color intensity represents the relative amount of the nucleotide base numbers of the genes containing cognate protein family (heavy: more similarity; moderate: intermediate similarity; light: less similarity). A range of BLAST hits were found to be classified into the same protein family. Notably, identified conserved and diverse genes are labeled with matching signatures; white: no hits; oblique stripes: relatively conservative; horizontal stripes: absolutely specific; others: absolutely conservative.

In order to determine the detailed QS system in *Pseudoalteromonas*, over 200 references were collected from ‘PubMed’ and ‘Google Scholar’ using the search terms ‘Quorum sensing’ and ‘Quorum sensing & *Pseudoalteromonas*’, and by capturing QS-related citations within these academic sources (Supplementary Table S1). All references were manually curated for details of QSs and/or their regulation targets. Overall, 67 QS regulators were collected from the following experimentally-validated information and KEGG-provided retrievals. Our studies also included the relevant data on regulation factors which have been well studied, such as the RetS/LadS regulatory system of *Pseudomonas* and LuxR-based sensing mechanisms in *Vibrio*. Then, the conserved domains were carefully curated using COG-based RPS-BLAST [25] and Pfam-based HMMER [26] programs to acquire their conserved protein domains; thereby, 27 assignments were provided to 67 regulatory elements (22/27 present in *Pseudoalteromonas*) (Figure 1). Lastly, the regulatory targets were collected and the activator/repressor information was accommodated in order to acquire and encompass a reservoir of the corresponding genetic contexts (Supplementary Table S1).

In accordance with *Pseudoalteromonas* phylogeny described using CVTree3, QS positive and negative regulator disseminations were found to exhibit comparable conservation (Figure 1). Interestingly, phylogenetic neighbors likely exhibit similar QS regulation patterns. Additionally, strains carrying stronger QS-based traits commonly exhibit similar QS regulatory architectures. The observation from T1lg65 and T1lg88 showed that QS-associated traits are related to regulator numbers and their disseminations. A variety of regulators were also found to localize on ‘genomic instable regions’ (shadow in blocks in Figure 1, indicating uniqueness in these strains). Lastly, *Pseudoalteromonas* heterogeneity fully indicated that regulators pertaining to different clades could simultaneously and/or sequentially influence QS-associated traits.

**Figure 2:**
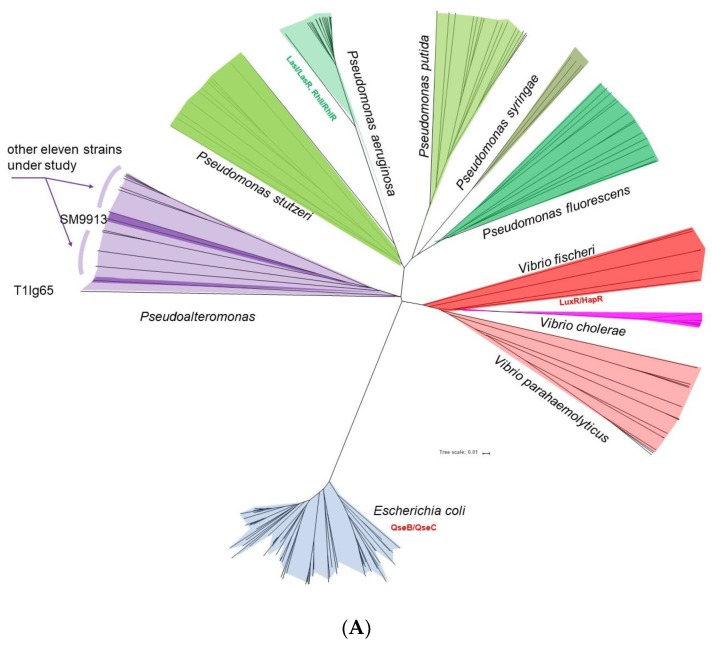

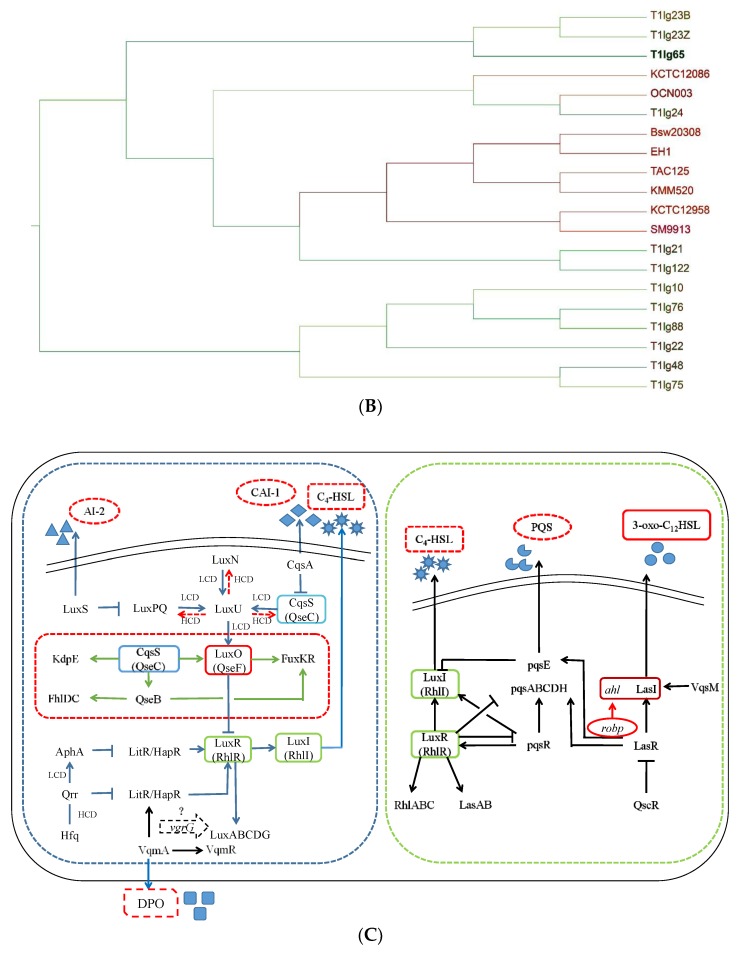
Evolutionary relationship between bacterial representatives with well-studied quorum sensing regulon and quorum sensing working model proposed from *Pseudoalteromonas*. (**A**) Schematic presentation of evolutionary relationship among *Pseudoaltermonas*, *Vibrio*, *Pseudomonas,* and *Escherichia coli* based on phylogenetic analysis of concatenated protein-coding sequences. Representative QS regulon in each clade was indicated if applicable: LuxR/HapR in *V.cholerae*V52 and *V. parahaemolyticus* RIMD 2210633; QseB/QseC in *E. coli* O157:H7; LasI/LasR in *P. aeruginosa* PAO1, *P. putid*a KT2440 and *P. syringae*pv. tomato strain DC3000. (**B**) *Pseudoalteromonas* phylogeny. Fully-sequenced *Pseudoalteromonas* strains in this phylogeny: *Pseudoalteromonas phenolica* KCTC 12086: chr1 (CP013187), chr2 (CP013188); *Pseudoalteromonas* sp. OCN003: chr1 (CP009888), chr2 (CP009889); *Pseudoalteromonas* sp. Bsw20308: chr1 (CP013138), chr2 (CP013139); *Pseudoalteromonas aliena* EH1: chr (CP019628); *Pseudoalteromonas haloplanktis* TAC125: chr1 (CR954246), chr2 (CR954247); *Pseudoalteromonas translucida* KMM 520:chr1 (CP011034), chr2 (CP011035); *Pseudoalteromonas issachenkonii* KCTC 12958: chr1 (CP013350); chr2 (CP013351); *Pseudoalteromonas* sp. SM9913: chr1 (CP001796); chr2 (CP001797). (**C**) Quorum sensing working model proposed from *Pseudoalteromonas*. The blue panel on the left in the working model schematics shows the QS system in *Vibri*o [11,12,33,34,35,36,37,38,39], whereas the red panel for that of *E. coli* [12,33]*.* The large light green dotted box presents the QS regulators in *Pseudomonas aeruginosa* [40,41,42,43]. Arrows indicate positive regulation (activation) and T-bars indicate negative regulation (inhibition). Red dotted arrows indicate positive regulation at high cell densities. Triangle represents signal molecule AI-2, diamond represents signal molecule CAI-1, and polygon represents signal molecule C4-HSL, together with 3/4 donut and droplet indicating signal molecule PQS and 3-oxo-C_12_-HSL, respectively (molecules with dotted line indicating non-AHL signals). Box represents signal molecule DPO (3,5-dimethylpyrazin-2-ol).

Besides the AHL-based colorization of A136 (Table 1), the inferred clade and candidate regulation factors in T1lg65 are obviously dissimilar to those from other isolates (Figure 1). Therefore, all identified QS genes of T1lg65 were reciprocally aligned with the other 12 *Pseudoalteromonas* genomic sequences. QS-related genes in T1lg65 were classified into three groups (Supplementary Table S2): absolutely conserved genes well-matched with other 12 genome sequences, non-conserved genes partially aligned, and completely unique genes unaligned with any other sequenced genomes (Supplementary Table S2). Similar results were obtained in T1lg88 as well (Supplementary Table S2). Moreover, the results denoted that most of QS related genes in T1lg65 are specific genes compared to other *Pseudoalteromonas* isolates, which is consistent with acquired adaption/survival fitness from horizontal genetic transfer events [27]. Phylogeny of the 12 *Pseudoalteromonas* strains in this work and representatives of *Pseudomonas*, *Vibrio,* and *Escherichia coli* implied that *Pseudoalteromonas* species likely emerge from the fascinating transition modulation form among a wide range of QS regulon representatives (Figure 2A). However, the T1lg65 clade is distinctly related to other isolates (Figure 2B).

QS-related colorization in T1lg65, T1lg88, and T1lg24 was examined by using cross-feeding bioassay. The results in Supplementary Figure S4A showed that T1lg65 and T1lg88 respectively exhibit the strongest and the weakest QS-dependent colorization of A136, and T1lg24 has colorization comparable to that of T1lg65. Subsequently, two important regulator genes, *luxO* and *rpoN,* respectively considered as positive and negative regulator genes in *Vibrio* and *Pseudomonas* [28,29,30,31,32], were chosen for analysis of relative expression level in these three *Pseudoalteromonas* isolates. Supplementary Figure S4B and Supplementary Figure S4C showed that the relative expression of both *luxO* and *rpoN* in three isolates is obviously different. The relative expression levels of *luxO* are the highest, the lowest, and moderate in T1lg65, T1lg88, and T1lg24, respectively, consistent with the QS-based colorization traits (Supplementary Figure S4A). Most probably, LuxO positively regulates the QS trait in *Pseudoalteromonas* as well. In contrast, the overall expression levels of *rpoN* are the lowest, the highest, and moderate in T1lg65, T1lg88, and T1lg24, respectively, which is opposite to QS-based colorization (Supplementary Figure S4A). Most probably, RpoN also negatively regulates QS trait in *Pseudoalteromonas*. Our data collectively demonstrate that both LuxO and RpoN play similar roles in regulating the QS system in *Pseudoalteromonas* as those in *Vibrio* and *Pseudomonas* [30]. Moreover, our survey further reveals that the QS trait correlates with genetic heterogeneity in *Pseudoalteromonas*, which is implicated in their phylogenetics (Figure 1 and Figure 2A).

### 2.4. Complicated Intermediate Quorum Sensing Schema in Pseudoalteromonas

Comparative genomics was performed to acquire the genetic contexts of QS regulators, including their position in the regulatory interaction and conserved enzymatic domains of proteinaceous regulators. Several regulators were identified by BLAST search carrying the same functional conserved domains, such as LuxO:QseF [protein family ID: COG2204] and CqsS:QseC [protein family ID: COG0642] (Supplementary Table S1 and Figure 2C). The collected information was systemically curated by text mining and comparative genomics.

As a result, a QS working model in *Pseudoalteromonas* was proposed, and such a schematic module sufficiently reflects the complexity of QS regulation in *Pseudoalteromonas* (Figure 2C). The current status of prior knowledge and data mining on these elements reveals a complex bacterial lifestyle. The scheme likely consists of three independent and cross-linked QS regulation networks related to those found in *E*. *coli*, *Pseudomonas,* and *Vibrio*. Four characteristics were presented by this model: (1) there are more regulators than any other known QS networks; (2) a variety of chemical molecules can be sensed by receptor proteins that, in turn, reflects cell density; (3) sRNA-like and proteinaceous regulators are included, accounting for this complicated system; (4) surprisingly, such diversified systems seem to exhibit in considerably perfect order. Interestingly, the interconnected regulation patterns under high and low cell density were clearly presented. For example, LuxN positively regulates LuxU at low cell densities, and LuxU activates LuxQ at high cell densities. Therefore, LuxO-, LuxR-, and LuxI-like regulation factors are characterized as unique nodes of ‘intermediate’ regulation status for insight into *Pseudoalteromonas* adaptive cascades.

### 2.5. Expanding the Proposed Schema via Identification of robp Essentiality for QS-Dependent Phenomenon

To acquire essential genes accounting for the highly enhanced QS-related colorization, the Tn-seq-based pipelines were performed in strain T1lg65. In total, 14 mutants with null AHL-based colorization of A136 due to mini-Tn*10* insertion were identified (Supplementary Table S3). Overall, these mutants have similar patterns of cell growth as the wild-type, e.g., slow growth in the initial stage and fast growth in the middle stage, followed by slower growth in the later stage (Supplementary Figure S5). On the other hand, the disruption of genes causes differences in cell growth, such as the *ompA*::Tn mutant giving a slower growth and a lower final cell density than WT, and the *dmcp*::Tn mutant yielding a higher cell density than WT from 5 h. In general, all mutant strains show slower growth in the first 4–5 h, compared to WT (Supplementary Figure S5). Also, the disruption of genes (Supplementary Table S3) causes changes in biofilm formation and motility (Supplementary Figure S5). Even if there are no statistically-significant differences in biofilm formation ability, there is a clear trend. All mutants have lower abilities to form biofilms. Additionally, five mutants, i.e., *rhtA*::Tn, *marR*::Tn, *robp*::Tn, *dmcp*::Tn and *capB*::Tn lose abilities in terms of swimming motility (Supplementary Figure S5). Among identified genes from 14 mutants (Supplementary Table S3), *robp* is considered as a candidate gene for positive regulation of QS signal formation in T1lg65 (Supplementary Figure S6), since partial *robp* sequence-encoded protein domain belongs to COG2207-like family.

**Figure 3:**
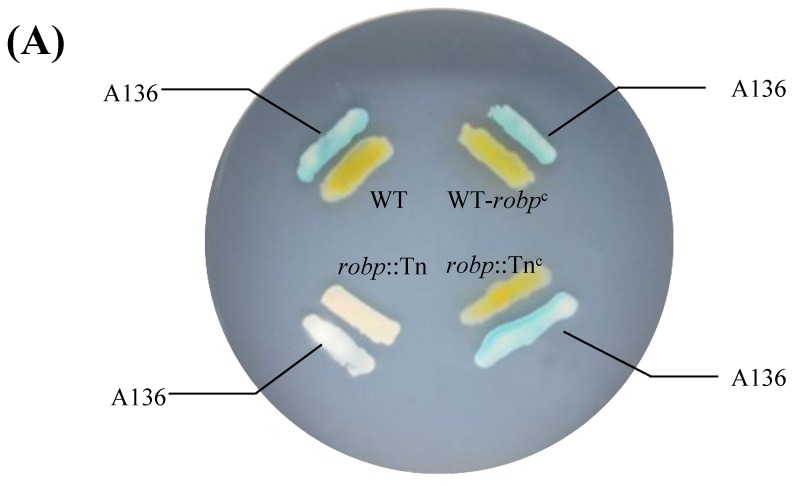

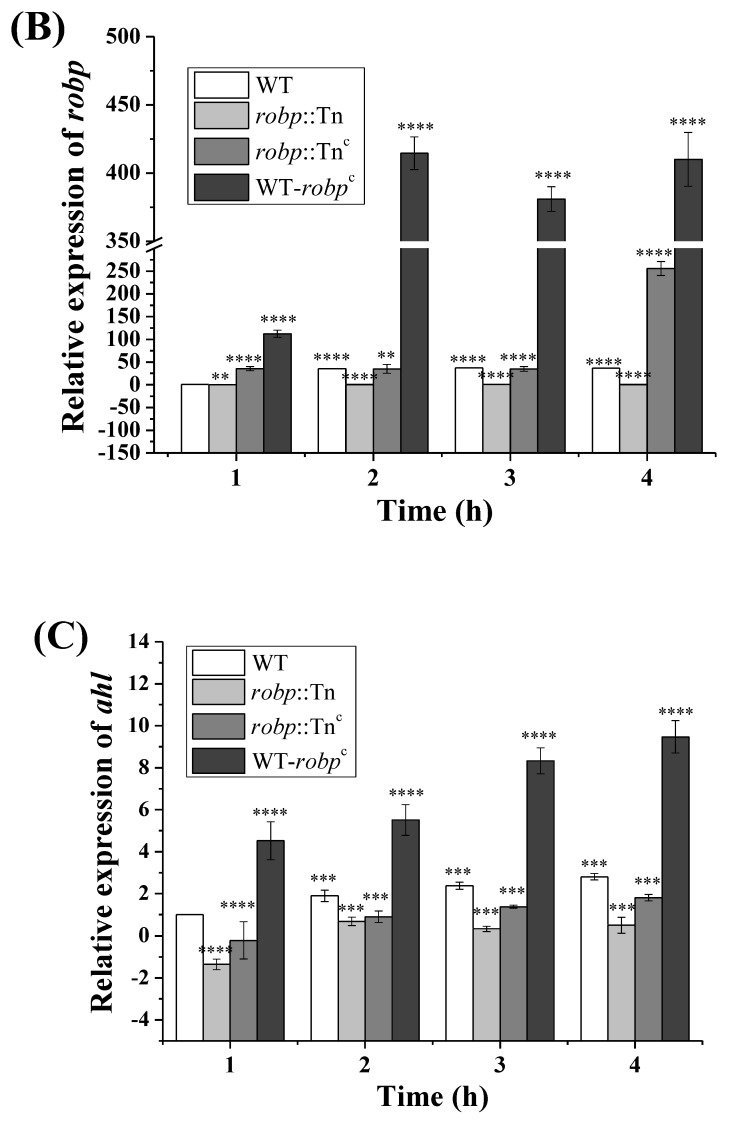
Growth profile and QS-related phenotype in wild-type, mutant, complicon and overexpressor. (**A**) Growth and QS-based colorization of biosensor A136; (**B**) Relative expression level of *robp* gene encoding right origin-binding protein; (**C**) Relative expression level of *ahl* gene encoding acyl homoserine lactone synthase. WT: wild-type (strain T1lg65); *robp*::Tn: mutant of strain T1lg65 with disrupted *robp* gene by Tn*10*; *robp*::Tn^c^: complon of *robp*::Tn with complementation of *robp*; WT+*robp^c^*: overexpressor of strain T1lg65 with overexpression of *robp*. All independent experiments were repeated in triplicate. Significant difference is respectively indicated by asterisks as follows: *** *p* < 0.001; **** *p* < 0.0001. Gene expression in WT at 1h was treated as 1 (internal reference).

In order to demonstrate the *robp* role to QS trait in T1lg65, four strains were prepared: wild-type (WT), mutant (*robp*::Tn, disruption of *robp* gene in strain T1lg65 by Tn*10*), complon (*robp*::Tn^c^, complementation of *robp* in *robp*::Tn), and overexpressor (WT+*robp^c^*, overexpression of *robp* in strain T1lg65). The growth and QS phenotype in WT, *robp*::Tn, *robp*::Tn^c^ and WT+*robp^c^* were accordingly compared. Figure 3A showed that the mutant (*robp*::Tn) yielded null colorization of A136. In contrast, the complon (*robp*::Tn^c^) generated almost completely restored colorization of A136, which is comparable to that from WT and overexpressor (WT+*robp^c^*). Our data further showed that the relative expression levels of both *robp* gene (Figure 3B) and *ahl* gene (Figure 3C) encoding acyl-homoserine lactone synthase (LasI in Figure 2) are overall high in WT+*robp^c^*, low in *robp*::Tn, and moderate in WT or *robp*::Tn^c^, indicating that *robp* is involved in positive regulation of QS signal formation in *Pseudoalteromonas*. Therefore, our bioinformatic analysis (Figure 1) and transposon mutagenesis mapping (Figure 3) congruously demonstrate that Robp serves as a positive regulator for QS system in *Pseudoalteromonas*.

**Figure 4:**
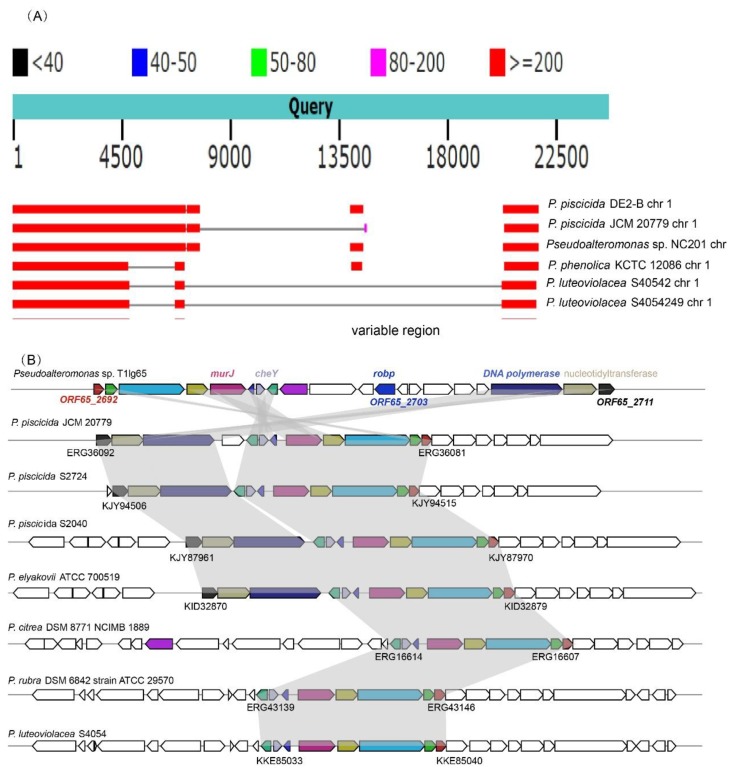
Region of genomic plasticity in the context of *robp*. (**A**) NCBI-BLAST outputs against RefSeq-archived sequences as of Jan, 2018 suggest that *robp* and neighboring genes could constitute a candidate genetic region of variability in *Pseudoalteromonas* strains, but its up-/down-stream genomic surroundings are comparably conserved; (**B**) The synteny alignment scheme indicates that *robp* is located in a region of genomic plasticity, indicating *robp* and its vicinity acquired by isolate T1lg65 are probably resulted from a series of genomic transposition and rearrangement. Genes coding for DNA polymerase, nucleotidyltransferase and Chemotaxis Y (CheY) protein are found to exist immediately adjacent to the ‘*robp* island’. Schematic diagrams of *robp* and its vicinity are drawn to scale.

Intriguingly, our further analysis in Figure 4A showed that *robp* and its proximity localize on a large region of plasticity (labeled as ‘*robp* island’ in this work) [44] in *Pseudoalteromonas*. Of note, acquisition/loss of the ‘*robp* island’ needs to be further clarified, since no other homologs were detected in other our sequenced genomes. Furthermore, the intact ‘*robp* island’ is also absent in RefSeq-archived sequences (Figure 4B). Such findings greatly outline the efficacy and important roles of horizontally acquired *robp* facilitating AHL-based QS (Supplementary Figure S6). Supplementary Figure S7 further shows that the backbone of genomes between T1lg65 and ATCC 700519 is nearly identical, but their genomic sizes and genetic architecture are different. In addition, no ‘*robp* island’ was found in ATCC 700519, indicating individual characteristics. Interestingly, no identical homolog was found in other strains. However, a variety of homologs (>1000) with <60% identities were predicted. They commonly contain AraC-like regulatory domains. For instance, Robp has some similarity to VqsM [45] in *P. aeruginosa* PAO1. These two protein sequences both include COG2207- or AraC-like domains (Supplementary Table S1). However, another domain is obviously different (Robp: GryI; VqsM: Arabinose_bd). In addition, the position of AraC encoding fragment in their sequences are different as well. This finding expands a wider landscape of functionality for AraC family regulators on quorum sensing.

## 3. Discussion

*Pseudoalteromonas* species are commonly isolated from different marine environments, such as the Arctic, California, Antarctica, and China’s Chukchi Sea. The ORF number of previously-reported *Pseudoalteromonas* genomes ranges from 3612 to 5012, with an average G+C content of 41% (generally ranging from 38% to 47%, data from NCBI). It has been reported that the morphological traits of *Pseudoalteromonas* are closely correlated with production/category of QS signal molecule [1,20,22]. In this study, QS-related traits and genome sequences of the 12 *Pseudoalteromonas* isolates were characterized in order to explore the association between individual heterogeneity and population behavior. It was found that their morphological behavior is different (Table 1 and Supplementary Figure S1). In addition, the 12 *Pseudoalteromonas* isolates are also different in ORF number and G+C content (Table 2). The genomic scale emphasizes a potential relationship between genomic variation and ecological population behavior in *Pseudoalteromonas*.

The regulatory schema (Figure 1 and Figure 2) indicated that diverse and elaborate interplays exist in QS regulatory system of *Pseudoalteromonas*, including regulator number and dissemination conservation/diversity. The presence of QS genes in the *Pseudoalteromonas* isolates varies greatly. The copy number variation and transmission of QS-related genes mainly constitute the genetic description of such high complexity. Among the 12 *Pseudoalteromonas* isolates, T1lg65 and T1lg88 exhibit the strongest and null AHL-based colorization, respectively. One plausible explanation is that T1lg65 and T1lg88 harbor more and less QS-related specific genes, respectively (Supplementary Table S2). Therefore, QS in *Pseudoalteromonas* probably correlates with genomic heterogeneity.

*Pseudoalteromonas* has previously been phylogenetically related with *Vibrio* and *Pseudomonas*. Our comparative genomics analysis dissects that the QS-related scheme and working model in *Pseudoalteromonas* (Figure 1 and Figure 2) are more complicate than that in *Vibrio*, *Pseudomonas,* and *Escherichia coli* [14,33,46,47]. QS in *Pseudoalteromonas* is likely to be a transitional form comprised of that in *Vibrio* and *Pseudomonas*. *Pseudoalteromonas* are commonly considered to have special metabolic capacities [48,49]. Metabolic regulation in *Pseudoalteromonas,* as well as cell individual growth/replication, is related to cell density [9]. *Pseudoalteromonas* species seem to achieve elaborate cell physiology through a sophisticated QS regulation system, and thus, facilitate adaption advantages to various complex niches. Emerging studies on non-AHL signal molecules are required to provide insights into QS-related lifestyle and persistence/fitness of *Pseudoalteromonas*.

Diversified QS systems in *Pseudoalteromonas* (Figure 1 and Figure 2) could bridge the huge knowledge gap between *Pseudoalteromonas* ‘mobilome’ and physiology-associated traits. Sixteen repeat regions were found in the T1lg65 genome, and this finding was not observed in other selected strains. Repeat regions could be closely related to horizontal gene transfer and genetic duplication-driven overexpression. Our transposon mutagenesis has mapped 14 QS associated genes, including *robp* and the type-VI secretion system associated *vgrG* [50,51] (Supplementary Table S3). However, only *robp* was found to share similarity to the bioinformatically-recruited, COG2207-like family, with positive regulatory role in QS system of *Pseudoalteromonas* (Figure 4), while the statuses of the remaining thirteen genes (Supplementary Table S3) are yet to be clarified, pointing out that QS system in *Pseudoalteromonas* is more complicated than what was originally thought. The *robp* flanking genes localize on a large region of plasticity (‘*robp* island’: potential implications for the spread of *robp*) in T1lg65 (Figure 4A) and no other homologs were detected in other *Pseudoalteromonas* genomes (Figure 4B), emphasizing the efficacy and important roles of horizontally-acquired *robp* facilitating AHL-based QS (Supplementary Figure S6). The OmpA family proteins are closely related to diversified cell communication, such as peptidoglycan-binding function and bacterial protective efficacy against adversity [51,52]. In addition, a putative *capB*-encoded capsular protein responds for bacterial invasion ability in *Francisella* [53]. It is possible that they both also contribute to QS-driven changes. Therefore, the newly-identified QS-associated patterns involving genetic conservation and variation significantly extend the repository of diversified mobilome-bearing adaptive and auxiliary determinants in *Pseudoalteromonas*.

## 4. Methods

### 4.1. Strains, Plasmids and Media

Zobell 2216E medium (sea salt 30g/L, tryptone 5 g/L, yeast extract 1 g/L, pH 7.6~7.8, agar 1.5~2% for solid) was used to culture the 12*Pseudoalteromonas*isolates collected from the Mangrove forest sediments in Ximen Island (28°20′33′′N, 121°10′28′′E), Zhejiang Province, China. Unless otherwise stated, *Pseudoalteromonas* isolates were stored in our lab at Zhejiang University of Technology and grew at 28°C overnight. In addition of morphological traits (Table 1), the taxonomy of all *Pseudoalteromonas* isolates was characterized based on the comparison of 16S rRNA gene sequence between 27F:5′AGAGTTTGATCCTGGCTCAG3′ and 1492R: 5′GGTTACCTTGTTACGACTT3′ (Table 1). All *Escherichia coli* and plasmids used in this study were listed in Supplementary Table S3. Unless otherwise specified, *E*. *Coli* cells grew in Luria-Bertani (LB) medium (NaCl 10 g/L, tryptone 10 g/L, yeast extract 5 g/L, agar 1.5~2% for solid) at 37°C overnight. If necessary, antibiotics were added to final concentrations as below: ampicillin (Amp) 100 μg/mL, tetracycline (Tet) 40 μg/mL, gentamycin (Gm) 50 μg/mL and kanamycin (Km) 50 μg/mL.

### 4.2. Cross-Feeding Bioassay

AHL-based QS traits of *Pseudoalteromonas* isolates were characterized by cross-feeding bioassay [54,55]. *Ensiferadhaerens* X097 was used as positive control in cross-feeding bioassay because it can produce AHLs, resulting in a blue color of indicator/biosensor *Agrobacterium tumefaciens* A136 [56]. A136 carries a fused *lacZ*-*traI*. AHLs with acyl side chain length from C_6_ to C_14_ can induce the expression of *lacZ* in A136 and 5-bromo-4-chloro-3-indolyl-b-D-galactopyranoside (X-gal) will be accordingly hydrolyzed to generate a blue color [54]*.*

Of note, A136 grows well on the LB medium, but not on the Zobell 2216E medium, whereas *Pseudoalteromonas* isolates grow well on Zobell 2216E medium. Therefore, cross-feeding bioassay for QS trait of *Pseudoalteromonas* isolates was performed on solid LB-2216E medium (obtained by mixing equal amounts of LB and Zobell 2216E) [54,57]. In general, after cultivation at 30°C for 12 h, colorization intensity of A136 [55] was recorded to describe the AHL-driven QS trait of *Pseudoalteromonas* isolates.

### 4.3. Genomic Sequencing and Assembly/Annotation

Single colony was picked from 2216E plate, and then transferred into shaking flask for overnight cultivation. Next, genomic DNA was extracted using a bacterial genomic DNA extraction kit (GE, Shanghai, China) for sequencing (Illumina Hi-Seq 2500 system, Vazyme Biotech Co.,Ltd, Nanjin, China). Lower quality sequencing data were filtered for accurate assembly [58]. The reads with average 500-bp were assembled using Velvet short sequence assembly software based on the *de-Bruijn* algorithm [59].

PROKKA [60] was used for bacterial gene prediction. The protein sequences of predicted genes were compared with NR [61], Swiss/TrEMBL [62], COG [63], and GO [64] databases to obtain the annotated information. Genomic rRNA and tRNA were predicted by using RNAmmer-1.2 [65] and tRNAscan-SE [66], respectively.

### 4.4. Comparative Genomics and Pan-Genomic Characterization

All-against-all BLASTn-based alignment was performed to define the core genomes. Conserved and auxiliary genes were characterized by given cut-offs (BLASTn identity ≥ 25, *E*-value ≤ 0.01 and matching length ≥ 100 bp). The core proteome phylogeny of the 12 *Pseudoalteromonas* isolates and previously-sequenced *Pseudoalteromonas* sp. SM9913 were therefore calculated and inferred by using CVTree3 [67]. To further illustrate the gene pools of *Pseudoalteromonas* species, the VENN scheme was provided to describe the relationship of selected distantly-related *Pseudoalteromonas* isolates.

### 4.5. Quorum Sensing Regulatory and Functional Gene Classification

Manual literature mining for QS regulators and their targets were performed with a range of associated keywords. To obtain accurate and detailed regulatory repertoire, related life process information from collected literatures was extracted and collated. In summary, four categories of regulatory data were acquired: (1) regulation factors, (2) downstream binding target, (3) successive activation/repression, and (4) nucleotide and protein sequence if available.

### 4.6. Transposon Mutagenesis

To create a mutant library with altered AHL-based colorization, transposon mutagenesis [68,69,70] was applied in tetracycline-resistant *Pseudoalteromonas* sp. T1lg65 with the strongest AHL-based colorization of A136 among isolates (Table 1). First, strain T1lg65 was cultured in 2216E broth. Meanwhile, *E. coli* S17-1(λpir) carrying suicide vector pLOF/Km with mini-Tn*10* was incubated in LB broth. After overnight growth, each culture was inoculated into fresh medium without any antibiotics, and reached an exponential growth phase. Second, 50 μL of T1lg65 receptor cells were spotted onto LB-2216E. After slight drying, another 50 μL of S17-1(λpir) donor cells were added onto T1lg65 cells. Negative controls with only S17-1(λpir) or T1lg65 were also prepared. After overnight conjugation, the cells were scraped and suspended in 1 mL of 2216E. After appropriate dilution, the cell mixture was spread onto 2216E with antibiotics for incubation. Finally, the target mutants with null AHL-based colorization of A136 were determined using cross-feeding bioassay [54,56].

### 4.7. Characterization of Transposon-Inserted Gene

Genomic DNA was prepared from Tn*10*-inserted mutant using a bacterial genomic DNA extraction kit (GE, USA). The Tn*10*-inserted DNA sequence in each mutant was characterized using high-efficiency thermal asymmetric interlaced PCR (hiTAIL-PCR) [70]. The primers for hiTAIL-PCR are listed in Supplementary Table S4. The first round PCR was conducted in a 20 μL system containing 20 ng DNA, 50 nM DTn10AP1, 1.0 μM of each LAD primer [70], 200 μM dNTPs, 2.0 μL of PCR buffer, and 2.5 U rTaq polymerases (TaKaRa, Dalian, China). Next, second round PCR was employed in a 50 μL system with 1.0 μL of appropriately-diluted PCR product from first round, 5.0 μL of PCR buffer, 50 nM DTn10AP2, 1.0 μM of each LAD primer, 200 μM dNTPs, and 2.5 U rTaq polymerases. Finally, third round PCR was performed in a 50 μL system with 1.0 μL of appropriately-diluted PCR product from second round, 5.0 μL of PCR buffer, 50 nM DTn10AP3, 1.0 μM of each LAD primer, 200 μM dNTPs, and 2.5U rTaq polymerases. The amplicons from the second and third rounds were evaluated on 1.5% agarose gel and the desired fragments were purified (Qiagen, MD, USA) for TA cloning (TaKaRa, Dalian, China). After sequencing in Sangon Biotech (Shanghai, China), the disrupted gene sequence in each mutant was obtained. After alignment with the relevant gene on our previously-collected genome, the transposon-inserted gene was accordingly determined.

### 4.8. Complementation and Overexpression of the Disrupted Gene

To complement the disrupted *robp* genein mutant of *robp*::Tn*10* (Supplementary Table S3) with null AHL-based colorization of A136, the entire *robp* was amplified using paired primers (robpF:5′ATGGATCCTTACTCAATAGGCAAGTAAATATCGGT3′, with a *Bam*H I restriction site; robpR:5′ATGGTACCATGTCAAAATATCAAAAGCGGTTTA3′, with a *Kpn*I restriction site). *Pfu* DNA polymerase (Promega, Beijin, China) was used to amplify 867 bp of *robp*. Then, *Taq* DNA polymerase (TaKaRa, Dalian, China) was used to add 3’-adenine overhang. Next, the desired product was purified for TA cloning (TaKaRa, China). After *Kpn*I/*Bam*H I digestion, the gene was released, and subcloned into *Kpn*I/*Bam*H I digested pBBR1MCS-5 [70] to generate plasmid pBBR1MCS-5/*robp* for transformation into *E. coli* HB101. Finally, assisted by plasmid pBR2013 in *E. coli* DH5α [70], pBBR1MCS-5/*robp*in *E. coli* HB101was transferred into mutant strain *robp*::Tn*10* and wild type strain T1lg65 for complementation and overexpression of *robp* gene, respectively, through tri-parental conjugation.

Tri-parental conjugation was performed as previously reported [70]. First, mutant or wild-type (receptor) *E. coli* HB101 with pBBR1MCS-5/*robp* (donor) and *E. coli* DH5α with pRK2013 (assistor) were separately cultured in 2216E with 40 μg/mL Tet and 50 μg/mL Km, LB with 50 μg/mL Gm and LB with 50 μg/mL Km, respectively. Next, 2 mL of each culture cells at exponential growth phase were pelleted after centrifugation at 5000 g for 5 min. After washing once, each cell pellet was gently resuspended in 0.5 mL of LB-2216E and mixed together. After centrifugation and removal of supernatant, the cell mixture was completely resuspended again in 0.2 mL of LB-2216E and dropped to sterilized microporous membrane (size diameter 1 cm; aperture 0.45 μm) on LB-2216E plate for overnight conjugation at 30°C. Finally, the microporous membrane was washed in 3 mL of 2216E to remove the cells. After appropriate dilution, 50 μL of cell mixtures were plated onto 2216E containing necessary antibiotics. After incubation at 30°C, the target colonies were molecularly confirmed.

### 4.9. Quantitative real-Time PCR for Detectionof Relative Gene Expression

The cultured *Pseudoalteromonas* cells were pelleted, and the total RNA was next prepared using RNAiso Plus kit (TaKaRa, Dalian, China). RNA integrity was determined according to OD_260nm_/OD_280nm_ ratio and around 500 ng of RNA samples were amplified to cDNA using PrimeScript^TM^ RT Master Mix kit (TaKaRa, Dalian, China). After appropriate dilution, the reverse-transcribed cDNA was further amplified to target gene fragment using SYBR green Premix Ex Taq^TM^ kit (TaKaRa, Dalian, China) [71]. All the primers (Supplementary Table S4) were designed according to the genome sequences of selected *Pseudoalteromonas* isolates. The PCR amplification was performed on CFX Connect Real-Time System (Bio-Rad, Hercules, CA) with a protocol of one cycle of denaturation at 95°C for 10 min and 40 cycles of denaturation at 95°C for 15 s and annealing/elongation at 60°C for 30 s. The 116 bp of 16S rRNA gene fragment (Supplementary Table S4) was used as internal control. The relative expression of target gene was calculated as previously reported [70].

### 4.10. Analysis of Data Significance

Unless otherwise specified, triplicate reactions per experiment were performed. All the data were presented as mean ± standard error and statistical significance was analyzed based on one-way analysis of variance followed by the Dunnett’s post hoc test using StatView 5.0 software. Significant difference is respectively indicated by asterisks as follows: * *p* < 0.05; ** *p* < 0.01; *** *p* < 0.001; **** *p* < 0.0001.

### 4.11. Availability of Data and Materials

The annotated draft genomes of *Pseudoalteromonas* isolates under this study have been submitted to WGS database under NCBI Accession Numbers listed in Table 2. The remaining data that support the findings of this study are available from the corresponding author upon request.

## 5. Conclusions

AHL-based colorization intensity of biosensors induced by *Pseudoalteromonas* most likely correlates with QS regulators genetic heterogeneity within the genus, providing a significantly better understanding of QS regulation in *Pseudoalteromonas*. Comprehensive QS regulatory schema and a working model proposed in *Pseudoalteromona*s seem to phylogenetically include the network architectures derived from *Escherichia coli*, *Pseudomonas,* and *Vibrio*, emphasizing that the distinctly- and hierarchically-organized mechanisms probably target QS regulation in bacterial dynamic genomes. Our studies improve understanding of the landscapes of QS regulation in *Pseudoalteromonas* and bacterial capabilities of accommodating their adaptation fitness and survival advantages.

## Figures and Tables

**Table 1:** Colony morphology and cross-feeding bioassay for AHL-based quorum sensing phenomenon of Pseudoalteromonas*.

Strain	Similarity (%)	16S RNA Top-Hit Strain	Colony	Color	Size **	Morphology	Colorization Intensity ***	
T1lg10	97.80	*P. tetraodonis* IAM14160	moist	white	big	round	+	
T1lg21	98.73	*P. shioyasakiensis* SE3	moist	white	big	round	+++	
T1lg22	97.92	*P. shioyasakiensis* SE3	moist	white	big	round	+	
T1lg23Z	99.43	*P. byunsanensis* FR1199	moist	purple	small	round	++	
T1lg23B	99.22	*P. byunsanensis* FR1199	moist	white	small	round	++	
T1lg24	99.71	*P. spongiae* UST010723-006	moist	orange	quite small	round	+	
T1lg48	97.82	*P. aestuariivivens*DB-2	moist	white	big	round	+++	
T1lg65	99.15	*P. elyakovii* ATCC 700519	moist	yellow	small	round	++++	
T1lg75	97.91	*P. robra* ATCC 29570	moist	white	big	round	+	
T1lg76	97.92	*P. tetraodonis* IAM14160	moist	white	big	round	++	
T1lg88	97.79	*P. aestuariivivens* DB-2	moist	white	big	round	-	
T1lg122	97.92	*P. aestuariivivens* DB-2	moist	white	big	round	++	

* This table reports the main features of *Pseudoalteromonas*phenotypic characteristics assayed after 12 h culture. ** Relative size. *** +: colorization intensity from biosensor strain A136 which was used to sense the QS signal molecules of AHLs with acyl side chain length from C_6_ to C_14_.

**Table 2:** Genomic characteristics of twelve *Pseudoalteromonas i*solates in this study.

Strain	Contig	Length (bp)	Gene Number	(G+C)%	WGS Accession Number	NCBI BioProject	tRNA	rRNA	tmRNA	CDS *	Repeat Region	Pigmen-Tation
T1lg10	57	3,430,516	3128	49.46	PQBV00000000	PRJNA430922	82	1	1	3039	1	NO
T1lg21	90	4,664,131	4298	41.33	PQBX00000000	PRJNA430929	88	10	1	4199		NO
T1lg22	42	3,429,413	3155	49.43	PQBY00000000	PRJNA430930	82	5	1	3078		NO
T1lg23B	55	4,584,947	4063	43.24	PQBZ00000000	PRJNA430934	85	6	1	3961		NO
T1lg23Z	54	4,583,886	4039	43.23	PQCA00000000	PRJNA430936	86	8	1	3950		YES
T1lg24	40	4,692,206	4241	40.83	PQCB00000000	PRJNA430938	73	4	1	4163		YES
T1lg48	24	3,589,059	3293	49.89	PQCC00000000	PRJNA430939	82	6	1	3206		NO
T1lg65	3683 **	6,875,118	6145	46.49	PQBW00000000	PRJNA430940	143	29	-	6027	16	YES
T1lg75	78	3,654,576	3317	50.13	PQCD00000000	PRJNA430941	95	7	2	3213		NO
T1lg76	131	3,378,987	3084	49.63	PQCE00000000	PRJNA430942	97	7	1	2981	1	NO
T1lg88	42	3,400,396	3097	49.49	PQCF00000000	PRJNA430943	71	9	1	3019		NO
T1lg122	47	4,592,438	4184	41.4	PQCG00000000	PRJNA430944	91	7	1	4094		NO

* CDS: protein coding sequences. ** a number of sequencing replicates to acquire more quality of the T1lg65 genome.

## Supplementary Materials

Supplementary materials can be found at http://www.mdpi.com/1422-0067/19/11/3636/s1.

## Abbreviations

QS	quorum sensing
AHL	acyl-homoserine lactone
COG	cluster of orthologous groups of proteins
*robp*	right origin-binding protein-encoding gene
Tn	transposon
LB	Luria-Bertani
