# Supplementary material for: Dissemination of Genetic Acquisition/Loss Provides a Variety of Quorum Sensing Regulatory Properties in Pseudoalteromonas
Source: Int J Mol Sci. 2018 Nov 18;19(11):3636. doi: 10.3390/ijms19113636 (PMC6275029; doi:10.3390/ijms19113636)
Supplement: Supplementary file 1 [file ijms-19-03636-s001.zip › Supplementary table S1.pdf]

Table S1. Characterized genomic properties for QS regulation collected in this study.

No.	Regulator	Domain	Representative Gene	Significant hits[a]	Homology Gene	Matching region (bp)		E-value	Reference[b]
1	LuxS	COG1854	VF_0545	T1lg24	orf24_03931	414	430	0.49	(1, 2)
2	LuxP	COG1879	VF_0707	T1lg23B	orf23B_02065	293	316	0.087	(3)
3	LuxM	-[c]	VIBHAR_RS12950	-[d]		-	-	-	(4)
4	LuxN	COG0642	VCA0522	T1lg48	orf48_01736	1837	1887	2.01E-05	(5)
5	LuxU	-	VF_0938	T1lg24	orf24_03140	102	129	0.095	(6)
6	LuxO	COG2204	VF_0937	T1lg24	orf24_02805	490	927	2.20E-09	(7)
7	AphA	COG3700	b4055	T1lg48	orf48_00938	531	555	0.012	(8)
8	LitR	COG1309	VF_2177	T1lg10	orf10_01573	87	108	0.034	(9)
9	HapR	COG1309	VC395_0600	T1lg75	orf75_01519	100	138	0.13	(10)
10	LuxR	COG2771	VF_A0925	T1lg23Z	orf23Z_01985	510	546	0.2	(11)
11	CqsA	COG0156	VIBNI_B1204	T1lg24	orf24_03672	356	393	0.093	(12)
12	CqsS	COG0642	VIBNI_B1205	T1lg23B	orf23B_03605	1820	1869	0.046	(13)
13	LuxI	-	VF_A0924	-		-	-	-	(14)
14	LasI	COG3916	PA1432	T1lg22	orf22_01623	342	368	0.42	(15, 16)
15	LasR	COG2771	PA1430	T1lg22	orf22_01075	404	431	0.14	(16, 17)
16	RhlI	COG3916	PA3476	T1lg65	orf65_01458	411	438	0.19	(18)
17	RhlR	COG2771	PA3477	T1lg22	orf22_01223	292	315	0.041	(19)
18	PhnABCDEH	-	-	-		-	-	-	-
19	PqsR	-	-	-		-	-	-	(20)
20	PhzI	cl17182	Pvag_0811	T1lg10	orf10_00741	43	70	0.12	(21)
21	PhzR	COG2771	Pvag_0810	T1lg24	orf24_03677	643	665	0.057	(21)
22	QseC	COG0642	b3026	T1lg10	orf10_01082	777	801	0.022	(22, 23)
23	QseB	COG0745	b3025	T1lg48	orf48_02296	227	308	7.67E-05	(22, 24)

24	KdpE	COG0745	b0694	T1lg88	orf88_01834	139	179	6.05E-06	(25)
25	QseE	COG0642	ECSP_3500	T1lg10	orf10_01533	757	797	0.082	-
25	QseF	COG2204	ECSP_3498	T1lg48	orf48_02953	562	887	2.85E-20	(26)
26	SdiA	COG2771	b1916	T1lg23B	orf23B_03552	488	521	0.056	(27)
27	RpaI	COG1599	ABM4_RS08420	T1lg23Z	orf23Z_02992	1755	1784	0.053	-
28	RpaR	-	-	-		-	-	-	-
29	CinI	cl17182	RHE_CH02916	T1lg88	orf88_01462	368	396	0.46	(28)
30	CinR	COG2771	RHECIAT_CH0003072	-		-	-	-	(29)
31	TraI	cl26262	pHN7A8_094	-		-	-	-	(30)
32	TraR	COG2771	pTi_029	T1lg88	orf88_01685	160	180	0.14	(31)
33	TraM	pfam09228	pTi_028	-		-	-	-	(32)
34	RaiI	cl17182	PGA2_c03440	T1lg122	orf122_03002	23	47	0.014	-
35	RaiR	COG2771	PGA2_c03430	T1lg88	orf88_02280	97	117	0.14	-
36	RhiI	cl17173	RCFBP_mp20340	T1lg122	orf122_02694	482	506	0.019	(33)
37	RhiR	COG2771	pRL100172	T1lg24	orf24_01966	94	116	0.2	-
38	EsaI	cl17182	SOD_c00740	T1lg23Z	orf23Z_01077	382	402	0.17	-
39	EsaR	COG2771	SOD_c00730	T1lg48	orf48_03068	349	390	0.004	(34)
40	TofI	cl17182	LW46_p1002	T1lg48	orf48_00332	416	451	0.011	-
41	TofR	COG2197	LW46_p1001	T1lg88	orf88_02913	433	455	0.14	-
42	CviI	cl17182	CV_4091	T1lg10	orf10_02986	530	553	0.039	-
43	CviR	COG2197	CV_4090	T1lg10	orf10_02497	706	722	0.55	-
44	AvsI	COG3916	Avi_1889	76	orf76_01869	330	358	0.036	-
45	AvsR	COG2771	Avi_1890	T1lg88	orf88_00475	142	171	0.012	-
46	YspR	COG2771	YPO0985	T1lg23B	orf23B_03277	591	626	0.005	(35)
47	SolR	COG2771	BDGL_003038	T1lg23B	orf23B_00066	535	635	0.005	(36)
48	QscR	COG2771	PA1898	T1lg75	orf75_03190	660	683	0.019	(37, 38)

49	RpoQ	COG0568	VF_A1015	T1lg48	orf48_03072	253	359	2.01E-07	(39)
50	AmpR	COG0583	PA4109	T1lg23B	orf23B_02812	19	127	1.10E-05	(40)
50	LsrB	COG1879	b1516	T1lg122	orf122_03017	89	112	0.08	(41)
51	YneA	COG1879	STM4077	T1lg48	orf48_02613	119	156	0.005	(42)
52	RpoS	COG0568	PA3622	T1lg48	orf48_03072	371	632	1.04E-49	(43, 44)
53	LsrG	COG1359	b1518	T1lg22	orf22_00388	87	113	0.67	(45)
54	YpeR	COG2771	YPO2457	T1lg10	orf10_02365	75	99	0.2	(35)
55	YenR	COG2771	YE1599	T1lg10	orf10_00100	574	597	0.012	(46)
56	*orf*154	pfam09228	pSmeSM11b_p154	-		-	-	-	(47)
57	QteE	-	PA2593	-		-	-	-	(48)
58	LsrK	COG1070	b1511	-		-	-	-	(49)
59	MqsR	pfam15723	b3022	-		-	-	-	(50)
60	TqsA	COG0628	b1601	T1lg48	orf48_02299	786	978	5.53E-28	(51)
61	Vfr	COG0664	PA0652	-		-	-	-	-
62	VqsM	COG2207	PA2227	T1lg122	orf122_01705	275	305	0.27	(52, 53)
63	PqsE	COG0491	PA1000	T1lg65	orf65_05517	338	365	0.29	(54)
64	RpoN	COG1508	PA4462	T1lg48	orf48_00275	1132	1451	7.03E-54	(55, 56)
65	PprB	COG2197	PA4296	-		-	-	-	(57)
66	RsmA	COG1551	PA0905	T1lg65	orf65_00657	1	143	8.51E-25	(58)
67	MvaT	-	PA4315	T1lg88	orf88_01688	284	305	0.25	(59)

[a] significant hit was found most similar to the representative gene in this strain, and matching regions was indicated as well.

[b] -: collected from review literatures, not experimental citation.

[c] no significant hit against COG (http://www.ncbi.nlm.nih.gov/COG) and Pfam (http://pfam.xfam.org/databases).

[d] no significant hit.

REFERENCES

1. **Yang Y, Zhou M, Hou H, Zhu J, Yao F, Zhang X, Zhu X, Hardwidge PR, Zhu G.** 2014. Quorum-sensing gene luxS regulates flagella expression and Shiga-like toxin production in F18ab *Escherichia coli*. Canadian journal of microbiology **60:**355-361.
2. **Le KY, Otto M.** 2015. Quorum-sensing regulation in staphylococci-an overview. Frontiers in microbiology **6:**1174.
3. **Ruby EG, Urbanowski M, Campbell J, Dunn A, Faini M, Gunsalus R, Lostroh P, Lupp C, McCann J, Millikan D, Schaefer A, Stabb E, Stevens A, Visick K, Whistler C, Greenberg EP.** 2005. Complete genome sequence of *Vibrio fischeri*: a symbiotic bacterium with pathogenic congeners. Proceedings of the National Academy of Sciences of the United States of America **102:**3004-3009.
4. **Milton DL, Chalker VJ, Kirke D, Hardman A, Camara M, Williams P.** 2001. The LuxM homologue VanM from *Vibrio anguillarum* directs the synthesis of N-(3-hydroxyhexanoyl)homoserine lactone and N-hexanoylhomoserine lactone. Journal of bacteriology **183:**3537-3547.
5. **Higgins DA, Pomianek ME, Kraml CM, Taylor RK, Semmelhack MF, Bassler BL.** 2007. The major *Vibrio cholerae* autoinducer and its role in virulence factor production. Nature **450:**883-886.
6. **Ray VA, Visick KL.** 2012. LuxU connects quorum sensing to biofilm formation in *Vibrio fischeri*. Molecular microbiology **86:**954-970.
7. **Kimbrough JH, Stabb EV.** 2015. Antisocial luxO Mutants Provide a Stationary-Phase Survival Advantage in *Vibrio fischeri* ES114. Journal of bacteriology **198:**673-687.
8. **Leone R, Cappelletti E, Benvenuti M, Lentini G, Thaller MC, Mangani S.** 2008. Structural insights into the catalytic mechanism of the bacterial class B phosphatase AphA belonging to the DDDD superfamily of phosphohydrolases. Journal of molecular biology **384:**478-488.
9. **Fidopiastis PM, Miyamoto CM, Jobling MG, Meighen EA, Ruby EG.** 2002. LitR, a new transcriptional activator in *Vibrio fischeri*, regulates luminescence and symbiotic light organ colonization. Molecular microbiology **45:**131-143.
10. **Ball AS, Chaparian RR, van Kessel JC.** 2017. Quorum Sensing Gene Regulation by LuxR/HapR Master Regulators in *Vibrios*. Journal of bacteriology **199**.
11. **Antunes LCM, Ferreira RBR, Lostroh CP, Greenberg EP.** 2008. A mutational analysis defines *Vibrio fischeri* LuxR binding sites. Journal Of Bacteriology **190:**4392-4397.
12. **Turan NB, Chormey DS, Buyukpinar C, Engin GO, Bakirdere S.** 2017. Quorum sensing: Little talks for an effective bacterial coordination. Trac-Trends In Analytical Chemistry **91:**1-11.
13. **Eberl L, Riedel K.** 2011. Mining quorum sensing regulated proteins - Role of bacterial cell-to-cell communication in global gene regulation as assessed by proteomics. Proteomics **11:**3070-3085.
14. **Mandel MJ, Stabb EV, Ruby EG.** 2008. Comparative genomics-based investigation of resequencing targets in *Vibrio fischeri*: focus on point miscalls and artefactual expansions. BMC genomics **9:**138.

15. **Gould TA, Schweizer HP, Churchill ME.** 2004. Structure of the *Pseudomonas aeruginosa* acyl-homoserinelactone synthase LasI. Molecular microbiology **53:**1135-1146.
16. **Veselova MA.** 2010. [Quorum sensing regulation in *pseudomonas*]. Genetika **46:**149-158.
17. **Wang C, McPherson JR, Zhang LH, Rozen S, Sabapathy K.** 2016. Transcription-associated mutation of lasR in *Pseudomonas aeruginosa*. DNA repair **46:**9-19.
18. **Nelson LK, D'Amours GH, Sproule-Willoughby KM, Morck DW, Ceri H.** 2009. *Pseudomonas aeruginosa* las and rhl quorum-sensing systems are important for infection and inflammation in a rat prostatitis model. Microbiology **155:**2612-2619.
19. **Garg N, Manchanda G, Kumar A.** 2014. Bacterial quorum sensing: circuits and applications. Antonie van Leeuwenhoek **105:**289-305.
20. **Ilangovan A, Fletcher M, Rampioni G, Pustelny C, Rumbaugh K, Heeb S, Camara M, Truman A, Chhabra SR, Emsley J, Williams P.** 2013. Structural basis for native agonist and synthetic inhibitor recognition by the *Pseudomonas aeruginosa* quorum sensing regulator PqsR (MvfR). PLoS pathogens **9:**e1003508.
21. **Morohoshi T, Yamaguchi T, Xie X, Wang WZ, Takeuchi K, Someya N.** 2017. Complete Genome Sequence of *Pseudomonas chlororaphis* subsp. aurantiaca Reveals a Triplicate Quorum-Sensing Mechanism for Regulation of Phenazine Production. Microbes and environments **32:**47-53.
22. **Clarke MB, Sperandio V.** 2005. Transcriptional autoregulation by quorum sensing Escherichia coli regulators B and C (QseBC) in enterohaemorrhagic *E. coli* (EHEC). Molecular microbiology **58:**441-455.
23. **Xie W, Dickson C, Kwiatkowski W, Choe S.** 2010. Structure of the cytoplasmic segment of histidine kinase receptor QseC, a key player in bacterial virulence. Protein and peptide letters **17:**1383-1391.
24. **Milton ME, Allen CL, Feldmann EA, Bobay BG, Jung DK, Stephens MD, Melander RJ, Theisen KE, Zeng D, Thompson RJ, Melander C, Cavanagh J.** 2017. Structure of the Francisella response regulator QseB receiver domain, and characterization of QseB inhibition by antibiofilm 2-aminoimidazole-based compounds. Molecular microbiology **106:**223-235.
25. **Narayanan A, Paul LN, Tomar S, Patil DN, Kumar P, Yernool DA.** 2012. Structure-function studies of DNA binding domain of response regulator KdpE reveals equal affinity interactions at DNA half-sites. PloS one **7:**e30102.
26. **Reading NC, Rasko DA, Torres AG, Sperandio V.** 2009. The two-component system QseEF and the membrane protein QseG link adrenergic and stress sensing to bacterial pathogenesis. Proceedings of the National Academy of Sciences of the United States of America **106:**5889-5894.
27. **Shimada T, Shimada K, Matsui M, Kitai Y, Igarashi J, Suga H, Ishihama A.** 2014. Roles of cell division control factor SdiA: recognition of quorum sensing signals and modulation of transcription regulation targets. Genes to cells : devoted to molecular & cellular mechanisms **19:**405-418.
28. **Khan SR, Mavrodi DV, Jog GJ, Suga H, Thomashow LS, Farrand SK.** 2005.

Activation of the phz operon of *Pseudomonas fluorescens* 2-79 requires the LuxR homolog PhzR, N-(3-OH-Hexanoyl)-L-homoserine lactone produced by the LuxI homolog PhzI, and a cis-acting phz box. Journal of bacteriology **187:**6517-6527.

29. **Zheng H, Mao Y, Zhu Q, Ling J, Zhang N, Naseer N, Zhong Z, Zhu J.** 2015. The quorum sensing regulator CinR hierarchically regulates two other quorum sensing pathways in ligand-dependent and -independent fashions in Rhizobium etli. Journal of bacteriology **197:**1573-1581.
30. **Clark NJ, Raththagala M, Wright NT, Buenger EA, Schildbach JF, Krueger S, Curtis JE.** 2014. Structures of TraI in solution. Journal of molecular modeling **20:**2308.
31. **Tannieres M, Lang J, Barnier C, Shykoff JA, Faure D.** 2017. Quorum-quenching limits quorum-sensing exploitation by signal-negative invaders. Scientific reports **7:**40126.
32. **Lopez J, Salazar L, Andres I, Ortiz JM, Rodriguez JC.** 1991. Nucleotide sequence of the oriT-traM-finP region of the haemolytic plasmid pSU316: comparison to F. Nucleic acids research **19:**3451.
33. **Remenant B, Coupat-Goutaland B, Guidot A, Cellier G, Wicker E, Allen C, Fegan M, Pruvost O, Elbaz M, Calteau A, Salvignol G, Mornico D, Mangenot S, Barbe V, Medigue C, Prior P.** 2010. Genomes of three tomato pathogens within the *Ralstonia solanacearum* species complex reveal significant evolutionary divergence. BMC genomics **11:**379.
34. **Schu DJ, Scruggs JM, Geissinger JS, Michel KG, Stevens AM.** 2014. Acyl-homoserine lactone recognition and response hindering the quorum-sensing regulator EsaR. PloS one **9:**e107687.
35. **Rosso ML, Chauvaux S, Dessein R, Laurans C, Frangeul L, Lacroix C, Schiavo A, Dillies MA, Foulon J, Coppee JY, Medigue C, Carniel E, Simonet M, Marceau M.** 2008. Growth of *Yersinia pseudotuberculosis* in human plasma: impacts on virulence and metabolic gene expression. BMC microbiology **8:**211.
36. **Zhan Y, Yan Y, Zhang W, Yu H, Chen M, Lu W, Ping S, Peng Z, Yuan M, Zhou Z, Elmerich C, Lin M.** 2011. Genome sequence of *Acinetobacter calcoaceticus* PHEA-2, isolated from industry wastewater. Journal of bacteriology **193:**2672-2673.
37. **Oinuma K, Greenberg EP.** 2011. Acyl-homoserine lactone binding to and stability of the orphan *Pseudomonas aeruginosa* quorum-sensing signal receptor QscR. Journal of bacteriology **193:**421-428.
38. **Liang H, Deng X, Ji Q, Sun F, Shen T, He C.** 2012. The *Pseudomonas aeruginosa* global regulator VqsR directly inhibits QscR to control quorum-sensing and virulence gene expression. Journal of bacteriology **194:**3098-3108.
39. **Cao X, Studer SV, Wassarman K, Zhang Y, Ruby EG, Miyashiro T.** 2012. The novel sigma factor-like regulator RpoQ controls luminescence, chitinase activity, and motility in Vibrio fischeri. mBio **3**.
40. **Balasubramanian D, Kumari H, Jaric M, Fernandez M, Turner KH, Dove SL, Narasimhan G, Lory S, Mathee K.** 2014. Deep sequencing analyses expands the *Pseudomonas aeruginosa* AmpR regulon to include small RNA-mediated regulation of iron acquisition, heat shock and oxidative stress response. Nucleic acids research

42:979-998.
41. **Pereira CS, de Regt AK, Brito PH, Miller ST, Xavier KB.** 2009. Identification of functional LsrB-like autoinducer-2 receptors. Journal of bacteriology **191:**6975-6987.
42. **Taga ME, Semmelhack JL, Bassler BL.** 2001. The LuxS-dependent autoinducer AI-2 controls the expression of an ABC transporter that functions in AI-2 uptake in *Salmonella typhimurium*. Molecular microbiology **42:**777-793.
43. **Kayama S, Murakami K, Ono T, Ushimaru M, Yamamoto A, Hirota K, Miyake Y.** 2009. The role of rpoS gene and quorum-sensing system in ofloxacin tolerance in *Pseudomonas aeruginosa*. FEMS microbiology letters **298:**184-192.
44. **Tarassova K, Tegova R, Tover A, Teras R, Tark M, Saumaa S, Kivisaar M.** 2009. Elevated mutation frequency in surviving populations of carbon-starved rpoS-deficient *Pseudomonas putida* is caused by reduced expression of superoxide dismutase and catalase. Journal of bacteriology **191:**3604-3614.
45. **Touchon M, Hoede C, Tenaillon O, Barbe V, Baeriswyl S, Bidet P, Bingen E, Bonacorsi S, Bouchier C, Bouvet O, Calteau A, Chiapello H, Clermont O, Cruveiller S, Danchin A, Diard M, Dossat C, Karoui ME, Frapy E, Garry L, Ghigo JM, Gilles AM, Johnson J, Le Bouguenec C, Lescat M, Mangenot S, Martinez-Jehanne V, Matic I, Nassif X, Oztas S, Petit MA, Pichon C, Rouy Z, Ruf CS, Schneider D, Tourret J, Vacherie B, Vallenet D, Medigue C, Rocha EP, Denamur E.** 2009. Organised genome dynamics in the E*scherichia coli* species results in highly diverse adaptive paths. PLoS genetics **5:**e1000344.
46. **Kim Y, Chhor G, Tsai CS, Fox G, Chen CS, Winans NJ, Jedrzejczak R, Joachimiak A, Winans SC.** 2017. X-ray crystal structures of the pheromone-binding domains of two quorum-hindered transcription factors, YenR of *Yersinia enterocolitica* and CepR2 of *Burkholderia cenocepacia*. Proteins **85:**1831-1844.
47. **Murata T, Ohnishi M, Ara T, Kaneko J, Han CG, Li YF, Takashima K, Nojima H, Nakayama K, Kaji A, Kamio Y, Miki T, Mori H, Ohtsubo E, Terawaki Y, Hayashi T.** 2002. Complete nucleotide sequence of plasmid Rts1: implications for evolution of large plasmid genomes. Journal of bacteriology **184:**3194-3202.
48. **Liang H, Duan J, Sibley CD, Surette MG, Duan K.** 2011. Identification of mutants with altered phenazine production in *Pseudomonas aeruginosa*. Journal of medical microbiology **60:**22-34.
49. **Ha JH, Eo Y, Ahn HC, Ryu KS.** 2017. Increasing the soluble expression and crystallization of the *Escherichia coli* quorum-sensing protein LsrK. Acta crystallographica. Section F, Structural biology communications **73:**253-258.
50. **Gonzalez Barrios AF, Zuo R, Hashimoto Y, Yang L, Bentley WE, Wood TK.** 2006. Autoinducer 2 controls biofilm formation in *Escherichia coli* through a novel motility quorum-sensing regulator (MqsR, B3022). Journal of bacteriology **188:**305-316.
51. **Herzberg M, Kaye IK, Peti W, Wood TK.** 2006. YdgG (TqsA) controls biofilm formation in *Escherichia coli* K-12 through autoinducer 2 transport. Journal of bacteriology **188:**587-598.
52. **Dong YH, Zhang XF, Xu JL, Tan AT, Zhang LH.** 2005. VqsM, a novel AraC-type global regulator of quorum-sensing signalling and virulence in *Pseudomonas aeruginosa*. Molecular microbiology **58:**552-564.

53. **Liang H, Deng X, Li X, Ye Y, Wu M.** 2014. Molecular mechanisms of master regulator VqsM mediating quorum-sensing and antibiotic resistance in *Pseudomonas aeruginosa*. Nucleic acids research **42:**10307-10320.
54. **Folch B, Deziel E, Doucet N.** 2013. Systematic mutational analysis of the putative hydrolase PqsE: toward a deeper molecular understanding of virulence acquisition in *Pseudomonas aeruginosa*. PloS one **8:**e73727.
55. **Viducic D, Murakami K, Amoh T, Ono T, Miyake Y.** 2016. RpoN Modulates Carbapenem Tolerance in *Pseudomonas aeruginosa* through *Pseudomonas* Quinolone Signal and PqsE. Antimicrobial agents and chemotherapy **60:**5752-5764.
56. **Cai Z, Liu Y, Chen Y, Yam JK, Chew SC, Chua SL, Wang K, Givskov M, Yang L.** 2015. RpoN Regulates Virulence Factors of *Pseudomonas aeruginosa* via Modulating the PqsR Quorum Sensing Regulator. International journal of molecular sciences **16:**28311-28319.
57. **Bernard CS, Bordi C, Termine E, Filloux A, de Bentzmann S.** 2009. Organization and PprB-dependent control of the *Pseudomonas aeruginosa* tad Locus, involved in Flp pilus biology. Journal of bacteriology **191:**1961-1973.
58. **Marden JN, Diaz MR, Walton WG, Gode CJ, Betts L, Urbanowski ML, Redinbo MR, Yahr TL, Wolfgang MC.** 2013. An unusual CsrA family member operates in series with RsmA to amplify posttranscriptional responses in *Pseudomonas aeruginosa*. Proceedings of the National Academy of Sciences of the United States of America **110:**15055-15060.
59. **Castang S, Dove SL.** 2010. High-order oligomerization is required for the function of the H-NS family member MvaT in *Pseudomonas aeruginosa*. Molecular microbiology **78:**916-931.
