# Supplementary material for: Dissemination of Genetic Acquisition/Loss Provides a Variety of Quorum Sensing Regulatory Properties in Pseudoalteromonas
Source: Int J Mol Sci. 2018 Nov 18;19(11):3636. doi: 10.3390/ijms19113636 (PMC6275029; doi:10.3390/ijms19113636)
Supplement: Supplementary file 1 [file ijms-19-03636-s001.zip › Supplementary table S2.pdf]

Table S2. Quorum sensing genes in *Pseudoalteromonas* sp. T1lg65 and T1lg88 were classified into three groups: absolutely conserved genes, non-conserved genes and completely unique genes.

T1lg65 gene	Annotation	Conserved status a	Homolog	Identityb	Matching_Start	Matching_End
ORF65_00084	lysyl-tRNA synthetase	+	rpaI	89.286	1	324
ORF65_00135	hypothetical protein PTD2_07604	-	pqsE	100	2	182
ORF65_00182	hypothetical protein P20429_2847	-	litR	88.462	1	253
ORF65_00185	polysaccharide biosynthesis protein	*	traR	81.818	1	650
ORF65_00253	RNA polymerase subunit RpoD	+	rpoQ	68.372	1	326
ORF65_00257	hypothetical protein PpisJ2_18132	-	rpoQ	90.909	1	370
ORF65_00259	hypothetical protein PpisJ2_18142	-	yenR	95	58	222
ORF65_00365	hypothetical protein PpisJ2_04358	-	litR	95.238	1	826
ORF65_00520	GTP-binding protein Der	+	luxI	100	1	489
ORF65_00525	glycine dehydrogenase	+	accR	100	1	961
ORF65_00552	hypothetical protein	-	ypeR	100	1	136
ORF65_00569	putative signal transduction protein	-	hapR	90.909	1	278
ORF65_00580	3,4-dihydroxy-2-butanone 4-phosphate synthase	+	qseF	100	1	370
ORF65_00623	leucine-responsive regulatory protein	+	yspR	90.909	1	162
ORF65_00657	carbon storage regulator	+	rsmA	76.923	1	60
ORF65_00663	capsule biosynthesis protein CapB	-	raiI	100	7	1354
ORF65_00730	hypothetical protein PpisJ2_12748	-	luxN	92.593	16	332
ORF65_00760	glutathione-regulated potassium-efflux system protein KefB	+	rhiR	83.333	1	657
ORF65_00851	hypothetical protein PpisJ2_01706	-	rhiR	95.238	1	250

ORF65_00861	flagellar biosynthesis protein FlhB	-	luxI	90.909	1	376
ORF65_00876	sigma-54 dependent response regulator; regulation of polar flagellae expression	*	luxO	82.927	1	462
ORF65_00879	flagellar regulatory protein A	+	luxO	72.152	1	478
ORF65_00936	cold-active alkaline serine protease	*	yspR	100	63	590
ORF65_01001	two component, sigma54 specific, Fis family transcriptional regulator	+	luxO	75.806	1	450
ORF65_01027	putative phage related protein	-	qseB	90.909	15	670
ORF65_01124	dihydroxyacid dehydratase	-	luxI	100	1	591
ORF65_01169	hypothetical protein PpisJ2_11285	-	luxM	91.304	1	282
ORF65_01180	two-component response regulator	-	luxO	77.941	1	464
ORF65_01206	oligopeptidase, type B	+	avsI	86.207	7	683
ORF65_01291	toxin secretion, membrane fusion protein	-	yenR	95	1	414
ORF65_01305	partitioning protein A	+	tqsA	82.051	1	258
ORF65_01347	potassium transport protein	+	qseC	80.952	1	483
ORF65_01378	DNA excision repair enzyme subunit, with UvrBC	+	lsrK	91.304	1	940
ORF65_01408	sigma N (sigma 54) factor of RNA polymerase	+	rpoN	72.727	1	499
ORF65_01439	5-methyltetrahydrofolate--homocysteine methyltransferase	-	cqsA	83.784	1	454
ORF65_01458	autoinducer synthesis protein LuxI	+	rhlI	85.714	1	198
ORF65_01573	RNA polymerase sigma factor	-	rpoS	70.33	1	617
ORF65_01581	RNA polymerase factor sigma-32	-	rpoQ	88.889	1	285
ORF65_01604	linear pentadecapeptide gramicidin synthetase LgrD	-	luxU	90.909	73	2678
ORF65_01627	putative peptidase	-	esaR	100	1	672
ORF65_01639	sensory histidine kinase in two-component regulatory system with RstA	-	pqsE	90.909	11	407

**ORF65_01645**	**Signal transduction histidine kinase**	*	**cqsS**	**80.556**	**23**	**822**
**ORF65_01701**	**hypothetical protein PpisJ2_14515**	-	**vfr**	**90.909**	**19**	**169**
**ORF65_01776**	**hypothetical protein PpisJ2_21342**	-	**luxR**	**95**	**1**	**895**
**ORF65_01782**	**acetolactate synthase isozyme III large subunit**	*	**qseF**	**88.462**	**1**	**573**
**ORF65_01873**	**hypothetical protein ATW7_13543**	+	**phzR**	**100**	**1**	**109**
**ORF65_01904**	**hypothetical protein PpisJ2_06720**	-	**esaR**	**100**	**5**	**784**
**ORF65_02020**	**bacteriophage replication Protein A**	-	**phzR**	**91.304**	**4**	**1047**
**ORF65_02030**	**hypothetical protein P20495_1742**	-	**sdiA**	**91.667**	**1**	**297**
**ORF65_02108**	**von Willebrand factor A**	-	**rpaI**	**91.304**	**1**	**326**
**ORF65_02173**	**putative outer membrane protein with a TonB box**	-	**mqsR**	**90.909**	**1**	**1070**
**ORF65_02327**	**Zn-dependent hydrolase**	-	**ypeR**	**90.909**	**3**	**53**
**ORF65_02536**	**hypothetical protein Sden_2083**	-	**tofR**	**100**	**39**	**573**
**ORF65_02676**	**NADPH2:quinone reductase**	-	**rpoQ**	**100**	**1**	**311**
**ORF65_02742**	**hypothetical protein Ppis**	-	**luxP**	**95.238**	**1**	**107**
**ORF65_02830**	**extracellular solute-binding protein domain-containing protein**	-	**qseB**	**80.556**	**19**	**396**
**ORF65_02863**	**hypothetical protein PpisJ2_06975**	-	**qseF**	**95.238**	**1**	**894**
**ORF65_02890**	**N-acetylglucosamine-6-phosphate deacetylase**	-	**rhiI**	**95**	**3**	**368**
**ORF65_02921**	**response regulator receiver domain-containing protein**	-	**sdiA**	**81.25**	**1**	**499**
**ORF65_03009**	**hypothetical protein PpisJ2_08503**	+	**rsmA**	**100**	**1**	**345**
**ORF65_03084**	**sensor signal transduction histidine kinase**	-	**qseC**	**91.429**	**1**	**450**
**ORF65_03087**	**damselysin**	-	**rpoQ**	**88.462**	**1**	**561**
**ORF65_03136**	**response regulator (activator) in two-component regulatory**	-	**cqsA**	**91.304**	**1**	**231**

ORF65_03241	serine/threonine protein kinase	*	yenR	81.818	1	899
ORF65_03274	anaerobic nitric oxide reductase transcription regulator	*	luxO	86.667	9	345
ORF65_03360	peptidyl-prolyl cis-trans isomerase	-	luxU	91.667	1	430
ORF65_03366	transketolase	*	luxO	91.304	1	664
ORF65_03372	carbon starvation protein CstA	*	solR	83.871	1	561
ORF65_03403	acriflavin resistance protein	-	luxP	92	9	1022
ORF65_03404	AcrB/AcrD/AcrF family protein	*	luxI	92.308	1	1084
ORF65_03479	protease	+	aphA	85.185	1	482
ORF65_03506	response regulator with CheY-like receiver domain and winged-helix DNA-binding domain	*	kdpE	87.097	1	101
ORF65_03569	hypothetical protein PrubA2_05597	+	kdpE	91.304	1	174
ORF65_03594	integral membrane transport protein	-	tqsA	75	1	339
ORF65_03690	sensor protein	*	luxN	88.889	1	1094
ORF65_03703	response regulator receiver sensor signal transduction histidine kinase	-	cqsS	80.556	1	465
ORF65_03808	arginine succinyltransferase	+	ypeR	100	1	341
ORF65_03826	hypothetical protein PpisJ2_17532	-	luxS	95	1	190
ORF65_03877	zinc protease	-	rpoN	100	1	941
ORF65_03885	ubiquinone carrier protein	-	luxN	100	1	200
ORF65_03927	hypothetical protein PrubA2_20424	-	tqsA	91.304	10	385
ORF65_04061	hypothetical protein PpisJ2_19994	*	esaR	100	10	943
ORF65_04095	osmolarity sensor protein	-	rpoQ	86.667	1	433
ORF65_04133	response regulator/GGDEF domain protein	*	luxR	88	3	410
ORF65_04293	Fis family transcriptional regulator	*	luxO	89.655	1	402

ORF65_04315	SapC protein, putative	-	luxN	100	1	248
ORF65_04323	2OG-Fe(II) oxygenase	-	pqsE	100	1	273
ORF65_04356	hypothetical protein	-	rpoS	85.714	1	459
ORF65_04371	hypothetical protein MstaS_16734	-	vqsM	100	25	193
ORF65_04385	ABC transporter permease	-	avsR	95	2	72
ORF65_04391	binding-protein-dependent transport system inner membrane protein	*	qseF	91.304	1	269
ORF65_04414	type I restriction enzyme, R subunit	-	cviR	95	1	401
ORF65_04415	glutamate synthase large chain precursor	*	rsmA	90.476	1	377
ORF65_04444	iron-sulfur cluster-binding protein	-	mvaT	95.238	1	299
ORF65_04470	exonuclease I	*	rhlI	100	1	347
ORF65_04525	fhlA gene product	-	qseF	68.072	1	246
ORF65_04588	cation transporter	*	avsR	85.185	4	219
ORF65_04627	hydro-lyase, Fe-S type, tartrate/fumarate subfamily, subunit alpha	*	qseB	90.909	1	280
ORF65_04721	urea ABC transporter ATP-binding protein UrtD	*	tofR	88.462	1	113
ORF65_04757	lytic transglycosylase	-	rpoS	100	1	230
ORF65_04944	integral membrane ATPase	-	litR	90.909	51	116
ORF65_04964	recombination protein F	*	cviR	92.857	2	229
ORF65_04986	glutamate synthase, small subunit, putative	-	tofR	81.25	1	135
ORF65_05016	putative NAD-specific glutamate dehydrogenase	-	qscR	86.667	1	69
ORF65_05068	beta-lactamase	-	tofI	90.909	1	94
ORF65_05289	conserved hypothetical protein	*	luxP	80	1	121
ORF65_05494	hypothetical protein SL1157_0674	-	esaR	81.25	15	138

**ORF65_05517**	**extracellular solute-binding protein**	**-**	**pqsE**	**85.714**	**1**	**170**
**ORF65_05566**	**hypothetical protein**	**-**	**luxI**	**100**	**1**	**345**
**ORF65_05643**	**malate:quinone oxidoreductase**	*****	**solR**	**91.304**	**1**	**78**
**ORF65_05722**	**hypothetical protein MstaS_10342**	**-**	**accR**	**85.185**	**3**	**132**
**ORF65_05731**	**periplasmic chaperone LolA**	**-**	**yspR**	**90.909**	**2**	**133**
**ORF65_05988**	**sulfate transporter**	**-**	**rpoN**	**91.304**	**5**	**84**
**ORF65_06045**	**RpoD family RNA polymerase sigma factor**	**-**	**rpoS**	**74.265**	**1**	**45**
**ORF65_06067**	**hypothetical protein QWA_17715**	**-**	**avsI**	**95**	**2**	**80**
**ORF65_06092**	**FucP**	**-**	**isrK**	**91.304**	**8**	**62**

T1lg88 gene	Annotation	Conserved status [a]	Homolog	Identity[b]	Matching Start	Matching_End
ORF88_00021	cob(I)yrinic acid a,c-diamide adenosyltransferase	+	*esaI*	88	4	198
ORF88_00027	iron ABC transporter permease	+	*qscR*	91.667	3	329
ORF88_00079	phosphoserine phosphatase	+	*accR*	85.185	124	332
ORF88_00085	hypothetical protein P20495_2971	+	*qseC*	88.462	378	634
ORF88_00102	fadD gene product	+	*ampR*	100	1	521
ORF88_00106	peptidase S8/S53 subtilisin kexin sedolisin	+	*qseF*	85.185	208	503
ORF88_00153	iron superoxide dismutase	+	*rpoN*	88.889	1	194
ORF88_00156	glutamate synthase	+	*sdiA*	100	692	1173
ORF88_00156	glutamate synthase	+	*rpoN*	95	692	1173
ORF88_00232	adenosine diphosphate phosphatase	-	*rhiI*	100	4	253
ORF88_00272	hypothetical protein	*	*yspR*	100	30	310
ORF88_00276	hypothetical protein P20439_3615	+	*rhlI*	95	1	210
ORF88_00301	phosphoenolpyruvate carboxykinase	+	*rpoN*	100	8	511
ORF88_00315	protoheme IX farnesyltransferase 2	+	*ampR*	84.375	15	298

ORF88_00324	repressor LexA	+	*rsmA*	90.909	1	201
ORF88_00370	hypothetical protein PH505_ce00080	*	*tqsA*	89.655	1	296
ORF88_00392	sensory box protein	+	*qseF*	95	1051	1226
ORF88_00405	ATP-dependent helicase HepA	+	*ampR*	100	49	962
ORF88_00413	diadenosine tetraphosphatase (Ap4A hydrolase)	*	*qteE*	100	1	263
ORF88_00447	glycosyl transferase family protein	-	*rpoN*	95.238	19	360
ORF88_00458	hypothetical protein PSM_A2431	-	*rpoN*	85.185	50	230
ORF88_00475	hypothetical protein RNAN_1240	-	*rpoN*	100	1	128
ORF88_00488	glutamyl-Q tRNA(Asp) synthetase	+	*phzI*	85.714	4	268
ORF88_00559	RNA polymerase sigma factor	+	*rpoS*	70.748	256	612
ORF88_00560	50S ribosomal protein L11 methyltransferase	+	*qseB*	92	1	292
ORF88_00590	glutamate-5-semialdehyde dehydrogenase	+	*qteE*	100	1	415
ORF88_00677	hypothetical protein PTD2_07264	+	*lsrG*	94.737	1	177
ORF88_00683	type IV pilus assembly protein PilM	+	*traM*	90.476	5	354
ORF88_00690	primosomal protein N'	+	*rpoS*	80	28	749
ORF88_00691	hypothetical protein ParcA3_08381	+	*tqsA*	100	52	174
ORF88_00713	diguanylate cyclase	+	*tofR*	90.909	420	601
ORF88_00728	dihydrodipicolinate synthase	+	*luxU*	90.909	30	303
ORF88_00740	BadM/Rrf2 family transcriptional regulator	-	*luxU*	100	1	143
ORF88_00746	nitrogen availability sensory kinase	+	*luxN*	92	9	350
ORF88_00747	nitrogen regulation protein NR	+	*luxO*	67.188	1	463
ORF88_00771	sigma N (sigma 54) factor of RNA polymerase	+	*rpoN*	69.283	1	495
ORF88_00805	glutamine amidotransferase	+	*qseF*	100	10	200
ORF88_00865	hypothetical protein PTD2_06314	-	*tqsA*	88.889	81	178
ORF88_00888	type IV pilus assembly protein tapB	+	*ampR*	100	61	556
ORF88_00908	phosphomethylpyrimidine kinase/thiamin-phosphate pyrophosphorylase	-	*rpoN*	95	4	268

ORF88_00909	putative adenylyltransferase	+	*rpoN*	88	4	251
ORF88_00933	iron ABC transporter permease	+	*qseE*	100	7	543
ORF88_00940	dihydrolipoyllysine-residue acetyltransferase component of pyruvate dehydrogenase complex	+	*avsR*	100	201	629
ORF88_00942	hypothetical protein	*	*phzR*	88.889	18	119
ORF88_00976	bifunctional aspartokinase / homoserine dehydrogenase 1	+	*ypeR*	90.909	1	450
ORF88_00985	sensory histidine kinase in regulatory system	+	*qseE*	78.049	222	471
ORF88_00987	response regulator in two-component regulatory system	-	*qseF*	66.871	1	448
ORF88_01025	hypothetical protein Shal_1983	-	*litR*	95.455	1	876
ORF88_01077	serine protease, subtilase family protein	*	*luxR*	100	5	1126
ORF88_01106	LuxR family DNA-binding response regulator	-	*sdiA*	95	3	206
ORF88_01123	hypothetical protein VITU9109_19829	-	*kdpE*	82.857	6	224
ORF88_01160	Chain A, Crystal Structure Of The Nerve Agent	+	*yneA*	95.455	10	435
ORF88_01162	potassium transport protein	+	*tqsA*	83.333	1	482
ORF88_01173	hypothetical protein PspoU_15846	+	*rpoN*	95.238	1	157
ORF88_01210	FKBP-type peptidyl-prolyl cis-trans isomerase FkpA	+	*solR*	95	31	241
ORF88_01240	iron complex outermembrane recepter protein	-	*cqsA*	95	22	689
ORF88_01255	diaminopimelate epimerase	+	*kdpE*	100	3	276
ORF88_01373	bifunctional isocitrate dehydrogenase kinase/phosphatase protein	+	*rhlI*	95	3	564
ORF88_01378	RNase E	+	*rhiR*	95	1	491
ORF88_01391	thymidylate kinase	+	*mvaT*	90.476	1	204
ORF88_01406	dihydroorotase	+	*qseE*	100	5	346
ORF88_01411	N-acetylglucosamine-6-phosphate deacetylase	+	*tqsA*	85.185	3	370
ORF88_01462	hypothetical protein PpisJ2_20178	*	*cinI*	86.207	1	378
ORF88_01502	delta-aminolevulinic acid dehydratase	+	*qscR*	95	7	335
ORF88_01527	anaerobic nitric oxide reductase transcription regulator	-	*qseF*	78.947	10	529
ORF88_01555	hypothetical protein PSM_A1942	*	*aphA*	100	7	140

ORF88_01596	apolipoprotein N-acyltransferase copper homeostasis protein	+	*litR*	100	19	514
ORF88_01618	deoxyribodipyrimidine photolyase-like protein	+	*rhlR*	88.889	17	520
ORF88_01685	hypothetical protein ParcA3_04578	,	*traR*	95.238	5	268
ORF88_01688	DNA mismatch repair protein	+	*mvaT*	88.462	1	220
ORF88_01693	23S rRNA (uracil1939-C5)-methyltransferase	+	*cviI*	100	13	429
ORF88_01694	hybrid sensory histidine kinase BarA	+	*cqsS*	77.273	182	506
ORF88_01715	hydrophobe/amphiphile efflux-1 family protein	+	*yneA*	100	1	1024
ORF88_01731	aerobic respiration control sensor protein ArcB	+	*qseB*	95	263	507
ORF88_01739	oxaloacetate decarboxylase	+	*luxO*	95	1	474
ORF88_01762	DNA mismatch repair protein MutS	-	*rpoN*	88.889	10	863
ORF88_01763	RNA polymerase subunit RpoD	+	*rpoS*	81.818	21	322
ORF88_01799	lysyl-tRNA ligase	+	*luxO*	88.462	2	510
ORF88_01834	transcriptional regulatory protein qseB	+	*kdpE*	85.366	1	220
ORF88_01856	serine protease, subtilase family	*	*luxN*	100	1	1646
ORF88_01874	peptidase S8/S53 subtilisin kexin sedolisin	-	*lsrK*	88.889	210	464
ORF88_01904	hypothetical protein TERTU_3967	*	*rpoQ*	84.375	89	258
ORF88_01927	malate synthase G	-	*sdiA*	91.304	252	724
ORF88_01928	isocitrate lyase	+	*rpoN*	100	3	534
ORF88_01994	hypothetical protein P20495_3697	+	*qseE*	95.455	30	285
ORF88_01997	putative protease	+	*traI*	74.074	4	223
ORF88_02036	putative deoxyribodipyrimidine photolyase	+	*tofI*	95	1	461
ORF88_02060	sigma-54 dependent trancsriptional regulator	+	*luxO*	82.5	1	441
ORF88_02071	3-oxoacyl-acyl-carrier-protein	-	*ampR*	92.308	1	405
ORF88_02160	metallo-beta-lactamase	+	*cviI*	88.462	14	389
ORF88_02163	TPR repeat-containing protein VC_2164	+	*qseF*	88.889	4	481
ORF88_02177	excinuclease ABC subunit B	+	*rsmA*	100	1	670

ORF88_02214	EBP family response regulator	-	*qseF*	65.351	1	446
ORF88_02224	hypothetical transcriptional regulator	-	*esaI*	90.909	1	291
ORF88_02251	glutaminase	+	*rpaI*	89.286	1	306
ORF88_02279	two component LuxR family transcriptional regulator	*	*ampR*	88	9	198
ORF88_02280	nitrite reductase	-	*raiR*	95.238	45	308
ORF88_02329	cation efflux system protein cusA	-	*litR*	95	1	1036
ORF88_02338	hypothetical protein	*	*luxO*	90.909	7	176
ORF88_02339	peptidase S8/S53 subtilisin kexin sedolisin	-	*yneA*	81.579	7	379
ORF88_02361	tRNA-ms	+	*yneA*	90.909	4	183
ORF88_02364	mechanosensitive ion channel family protein	-	*esaI*	100	278	531
ORF88_02370	transporter	+	*luxU*	94.737	45	398
ORF88_02375	fumarylacetoacetase	-	*lsrG*	100	89	388
ORF88_02378	transcriptional regulatory protein tyrR	+	*luxO*	86.111	1	514
ORF88_02382	acyltransferase	+	*rhlR*	92	5	235
ORF88_02384	smr domain (Small MutS Related) containing protein	+	*lsrB*	100	9	186
ORF88_02386	chemotaxis protein	+	*kdpE*	77.273	6	118
ORF88_02403	type IV pilus biogenesis protein	+	*ypeR*	88	15	171
ORF88_02440	putative TonB-dependent receptor; outer membrane	+	*tofR*	91.304	355	996
ORF88_02445	N5-glutamine S-adenosyl-L-methionine-dependent methyltransferase	+	*qseF*	85.185	19	308
ORF88_02466	peptidase M75, Imelysin	-	*mvaT*	100	5	424
ORF88_02587	heat shock protein 90	*	*phzI*	90.909	4	633
ORF88_02629	UPF0118 membrane protein HI_0338	+	*rpoS*	100	9	337
ORF88_02631	two-component system, OmpR family, response regulator PhoP	+	*kdpE*	81.579	1	224
ORF88_02645	RNA binding protein	+	*cinI*	100	2	206
ORF88_02666	glucokinase	+	*yspR*	95	10	330
ORF88_02773	Carboxylesterase	+	*qseC*	100	1	282

ORF88_02787	isocitrate dehydrogenase (NADP)	-	*yenR*	96	2	740
ORF88_02913	desthiobiotin biosynthesis protein	+	*tofR*	91.304	6	133
ORF88_02958	sodium ABC exporter ATP-binding protein	+	*qseC*	100	32	263
ORF88_02982	Phosphorylase	+	*rhlR*	85.714	19	822
ORF88_02983	glycogen operon protein	+	*mvaT*	90.909	6	685
ORF88_03011	hypothetical protein PpisJ2_07040	*	*qseC*	92	2	924
ORF88_03036	cation transport ATPase, E1-E2	-	*qseE*	86.207	92	792
ORF88_03085	sigma-54 dependent transcriptional regulator/sensory box protein	+	*qseF*	91.304	1	473
ORF88_03087	hypothetical protein P20311_2860	-	*rpoN*	91.667	1	856

[a] Identified quorum sensing related genes of *Pseudoalteromonas* sp. T1lg88 were divided into absolutely conserved genes (+, orange), non-conserved genes (*, green) and specific genes (-, white).

[b] BLASTn generated results against customized database (Table S1).
