# Supplementary material for: Dissemination of Genetic Acquisition/Loss Provides a Variety of Quorum Sensing Regulatory Properties in Pseudoalteromonas
Source: Int J Mol Sci. 2018 Nov 18;19(11):3636. doi: 10.3390/ijms19113636 (PMC6275029; doi:10.3390/ijms19113636)
Supplement: Supplementary file 1 [file ijms-19-03636-s001.zip › Supplementary table S3.pdf]

Table S3. Bacterial strains and plasmids used in this study.

Strain or plasmid	Description	Resource
Escherichia coli		
S17-1(λpir)	Tp^r Sm^r, *recA ths hsdRM*$^+$, λpir phage lysogen RP4::Mu::Km Tn*7*	(60, 61)
DH5α	F^-, φ 80d*lacZ* ΔM15, Δ(*lacZYA -argF*)U169, *deoR* , *recA1* , *endA1* , *hsdR17* (*rK*$^-$, *mK*$^+$), *phoA*, *supE44* , *λ*$^-$, *thi -1*, *gyrA96* , *relA1*; host of plasmid pRK2013	Lab collection
HB101	*supE*44, △ (*mcr*C-*mrr*), *recA*13, *ara*-14, *proA*2, *lacY*1, *galK*2, *rpsL*20, *xyl*-5, *mtl*-1, *leu*B6, *thi*-1; host of plasmid pBBR1MCS-5	Lab collection
Pseudoalteromonas sp. T1lg65 and its derivatives		
T1lg65	Wild type; spontaneously resistant to tetracycline	This study
rhtA::Tn*10*	mutant with abolished QS; disrupted gene encoding threonine/homoserine exporter RhtA; Tet^rKm^r	This study
marR::Tn*10*	mutant with abolished QS; disrupted gene encoding MarR family transcriptional regualtor; Tet^rKm^r	This study
hyp1::Tn*10*	mutant with abolished QS; disrupted gene encoding hypothetical protein; Tet^rKm^r	This study
tonB::Tn*10*	mutant with abolished QS; disrupted gene encoding TonB-dependent receptor; Tet^rKm^r	This study
hyp2::Tn*10*	mutant with abolished QS; disrupted gene encoding hypothetical protein; Tet^rKm^r	This study
hyp3::Tn*10*	mutant with abolished QS; disrupted gene encoding hypothetical protein; Tet^rKm^r	This study
ompA::Tn*10*	mutant with abolished QS; disrupted gene encoding outer membrane protein A precursor; Tet^rKm^r	This study
*hyp*4::Tn*10*	mutant with abolished QS; disrupted gene encoding hypothetical protein; Tet^rKm^r	This study
robp::Tn*10*	mutant with abolished QS; disrupted gene encoding right origin-binding protein; Tet^rKm^r	This study
dmcp::Tn*10*	mutant with abolished QS; disrupted gene encoding HDOD domain-containing protein; Tet^rKm^r	This study
capB::Tn*10*	mutant with abolished QS; disrupted gene encoding capsule biosynthesis protein CapB; Tet^rKm^r	This study
lgsD::Tn*10*	mutant with abolished QS; disrupted gene encoding linear gramicidin synthase subunit D; Tet^rKm^r	This study
vgrG::Tn*10*	mutant with abolished QS; disrupted gene encoding VgrG protein; Tet^rKm^r	This study
hyp5::Tn*10*	mutant with abolished QS; disrupted gene encoding hypothetical protein; Tet^rKm^r	This study
Plasmids		
pLOF/Km	Ori R6K, mob RP4, Ap^r, mini-Tn*10* Km^r; suicide vector for transposon mutagenesis	(62)
pRK2013	Rep, LacZ, Gm^r; assistant plasmid tri-parental conjugation	(63)

pBBR1MCS-5	Derivative of IncP-1 plasmid RK2, *Tra*$^{+}$, ColE1, oriT, Km^{r}; delivery vector for complementation or overexpression of disrupted gene	(64)

References

60. **Huang YL, Li M, Yu Z, Qian PY.** 2011. Correlation between pigmentation and larval settlement deterrence by *Pseudoalteromonas* sp. sf57. Biofouling **27:**287-293.
61. **Solano F, Lucas-Elio P, Fernandez E, Sanchez-Amat A.** 2000. *Marinomonas mediterranea* MMB-1 transposon mutagenesis: isolation of a multipotent polyphenol oxidase mutant. Journal of bacteriology **182:**3754-3760.
62. **Herrero M, de Lorenzo V, Timmis KN.** 1990. Transposon vectors containing non-antibiotic resistance selection markers for cloning and stable chromosomal insertion of foreign genes in gram-negative bacteria. Journal of bacteriology **172:**6557-6567.
63. **Kovach ME, Elzer PH, Hill DS, Robertson GT, Farris MA, Roop RM, 2nd, Peterson KM.** 1995. Four new derivatives of the broad-host-range cloning vector pBBR1MCS, carrying different antibiotic-resistance cassettes. Gene **166:**175-176.
64. **Figurski DH, Helinski DR.** 1979. Replication of an origin-containing derivative of plasmid RK2 dependent on a plasmid function provided in trans. Proceedings of the National Academy of Sciences of the United States of America **76:**1648-1652.
