# Supplementary material for: Dissemination of Genetic Acquisition/Loss Provides a Variety of Quorum Sensing Regulatory Properties in Pseudoalteromonas
Source: Int J Mol Sci. 2018 Nov 18;19(11):3636. doi: 10.3390/ijms19113636 (PMC6275029; doi:10.3390/ijms19113636)
Supplement: Supplementary file 1 [file ijms-19-03636-s001.zip › Supplementary table S4.pdf]

Table S4. Oligonucleotide primers in real-time PCR and high-efficiency thermal asymmetric interlaced PCR.

**real-time PCR**			
**Strain [Gene]**	**Forward primer (5'-3')**	**Reverse primer (5'-3')**	**Product size (bp)**
T1lg24 [*luxO*]	GGCACCGGTAAAGAGCTGTG	TGGAATCGCCGCACAGTTTA	120
T1lg24 [*rpoN]*	ATGAAGCAATCTTTACAGCTTCGC	CGCTTCTTGAATTTCTTGCTGC	116
T1lg65 [*luxO]*	TGGCACTGGTAAAGAATTAGTGGC	GCCAGACTCTCAGGTAAAGATGC	118
T1lg65 [*rpoN]*	ATGAGACAATCATTACAGCTGCGT	TGCTTCTTGTATTTCTTGTTGTAGATCC	118
T1lg88 [*luxO]*	TGGTACGGGTAAAGAGCTGGTC	GAGCTCAGACTCCACCAAGTCC	118
T1lg88 [*rpoN]*	ATGAGGCAATCATTACAGCTGCG	CGCTTCTTGAATTTCTTGTTGGA	116
T1lg65 [*robp]*	AGCCAGTTAGAAGACAATGCAGACG	CCAATCGCATCATCGCTACCAATATG	118
T1lg65 [*ahl]*	ACTCAGTGTTGCAGCAGGCTTATC	CGTTCCATTGCTCTGGTGGTCAC	85
All [16S]	AAGAAGCACCGGCTAACTCC	TCTCGCTTAATCAACCGCCT	118

**high-efficiency thermal asymmetric interlaced PCR**	
primers[a]	Oligonucleotide sequences[b]
DTn10AP1	5'-TTGCCCGACATTATCGCGAGCCCAT-3'
DTn10AP2	5'-CAACACCTTCTTCACGAGGCAGACC-3'
DTn10AP3	5'-CGTTGCGCTGCCCGGATTACAGCCG-3'
LAD1-1	5'-ACGATGGACTCCAGAGCGGCCCGCVNVNNNGGAA-3'
LAD1-2	5'-ACGATGGACTCCAGAGCGGCCCGCBNBNNNGGTT-3'
LAD1-3	5'-ACGATGGACTCCAGAGCGGCCCGCHNVNNNCCAC-3'
LAD1-4	5'-ACGATGGACTCCAGAGCGGCCCGCVVNVNNNCCAA-3'
LAD1-5	5'-ACGATGGACTCCAGAGCGGCCCGCBDNBNNNCGGT-3'

[a] DTn10AP1, DTn10AP2 and DTn10AP3 are primers specific to transposon gene; LAD1-1, LAD1-2, LAD1-3, LAD1-4 and LAD1-5 are arbitrary primers.

[b] N=A/C/G/T; V=A/C/G; B= C/G/T; H=A/C/T; D=A/G/T.
