# Supplementary material for: Dissemination of Genetic Acquisition/Loss Provides a Variety of Quorum Sensing Regulatory Properties in Pseudoalteromonas
Source: Int J Mol Sci. 2018 Nov 18;19(11):3636. doi: 10.3390/ijms19113636 (PMC6275029; doi:10.3390/ijms19113636)
Supplement: Supplementary file 1 [file ijms-19-03636-s001.zip › ijms-376795 supplementary figures.pdf]

Supplementary S1: Biofilm formation assay using crystal violet

For each assay, 50 μL of glycerol cell stock, routinely stored at −80°C, was plated onto 2216E plate, and single colony was picked for inoculation in 5 mL of 2216E broth. Cultures were aerobically grown overnight with shaking at 30°C. The culture was diluted to around 1×10^9 CFU/mL in fresh broth, and 1 mL was added to a sterile glass test tube. Tubes were incubated for 72 h at 30°C without shaking. For crystal violet staining, cultures were removed. Next, tubes were washed with water for three times and then dried at 60°C for 30 min. One milliliter of a 1% crystal violet solution was added, and the tubes were incubated on a rocker at room temperature for 30 min. Unbound crystal violet was washed off with water, and the tubes were dried at 37°C. Bound crystal violet was dissolved into 1 mL of 33% glacial acetic acid and then poured into cuvettes. Finally, absorbance at 570nm was measured.

Reference: Wang PX, Zeng ZS, Wang WQ, Wen ZL, Li J, Wang XX. 2017. Dissemination and loss of a biofilm-related genomic island in marine *Pseudoalteromonas* mediated by integrative and conjugative elements. Environmental Microbiology 19:4620-4637.

Swimming motility assays

For each assay, a 50-μl single-use glycerol stock, routinely stored at −80°C, was plated onto a 2216E plate, and a single colony was picked for inoculation in 5 mL of 2216E broth. Cultures were grown aerobically with shaking overnight at 30°C. Next, cell cultures were standardized to an optical density at 600nm of around 0.6, and 0.5μL of sample was spotted onto the center of 2216E plates with 0.3% agar. After dry for 30 min at room temperature, the plates were incubated upright for 12 h at 30°C, and swimming zone diameters were quantified using vernier calliper.

Reference: Qin QL, Li Y, Zhang YJ, Zhou ZM, Zhang WX, Chen XL, Zhang XY, Zhou BC, Wang L, Zhang YZ. 2011. Comparative genomics reveals a deep-sea sediment-adapted life style of *Pseudoalteromonas* sp. SM9913. ISME J 5:274-284.

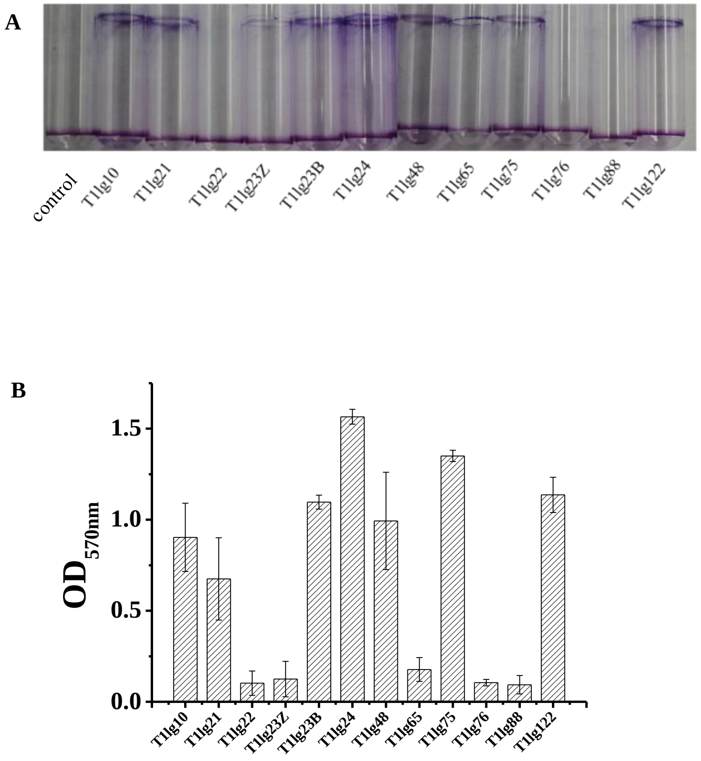

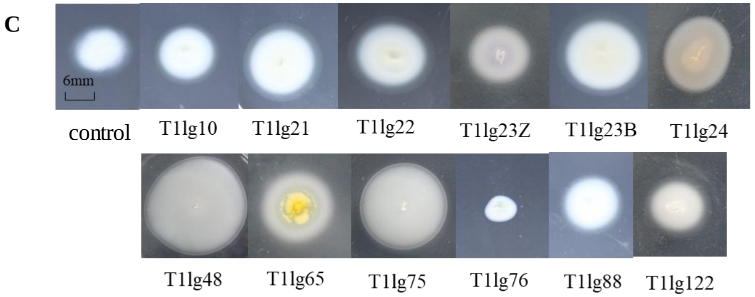

Strain	Motility Size (mm)
control	7
T1lg10	15
T1lg21	18
T1lg22	18
T1lg23Z	14
T1lg23B	22
T1lg24	22
T1lg48	38
T1lg65	12
T1lg75	18
T1lg76	6
T1lg88	12
T1lg122	12

Figure S1. The efficacy of biofilm formation and swimming motility of *Pseudoalteromonas* strains in this study. (A) Crystal violet staining was used to acquire biofilm formation efficacy in 12 isolated *Pseudoalteromonas* strains; (B) Biofilm formation was evaluated based on detection of bound crystal violet at OD_{570nm}; (C) Swimming motility of isolated *Pseudoalteromonas* strains under study. Control: *Shewanella oneidensis*.

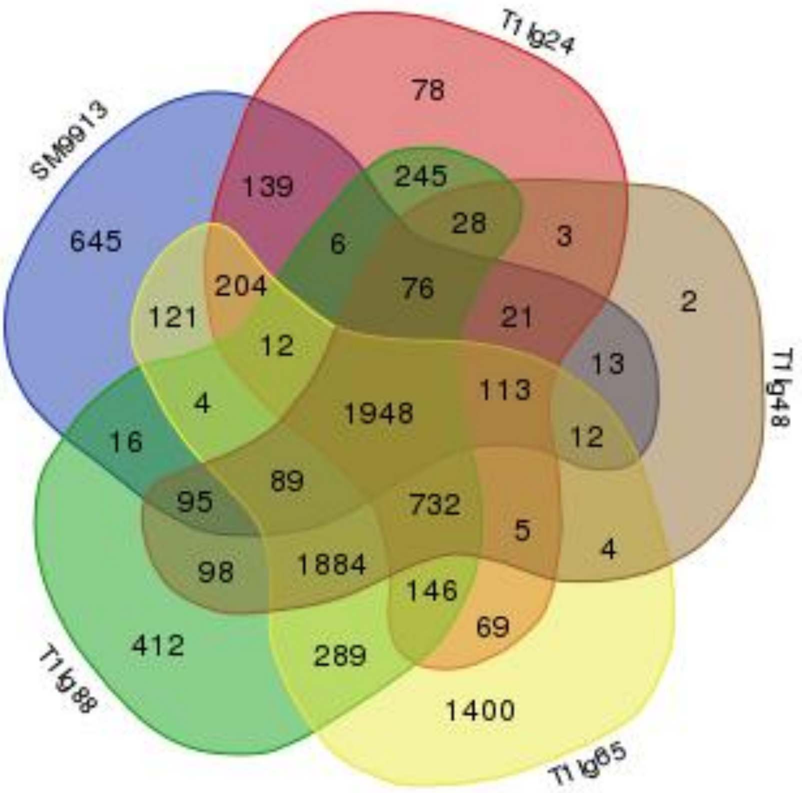

Figure S2. Venn diagram showing the conservative/specific relationship between all protein-coding genes of selected five *Pseudoalteromonas* representatives. The number of gene pertaining to each group was indicated. Blue: *Pseudoalteromonas* sp. SM9913; green: *Pseudoalteromonas* sp. T1lg88; red: *Pseudoalteromonas* sp. T1lg24; yellow: *Pseudoalteromonas* sp. T1lg65; brown: *Pseudoalteromonas* sp. T1lg48. QS-related traits of strain T1lg65 was stronger than the others, whereas no significant color change of biosensor A136 was observed for T1lg88. The Venn diagram showed that the selected *Pseudoalteromonas* representatives are significantly different in core and specific genes. T1lg65 has 1400 specific genes, whereas T1lg88 has 412 specific genes.

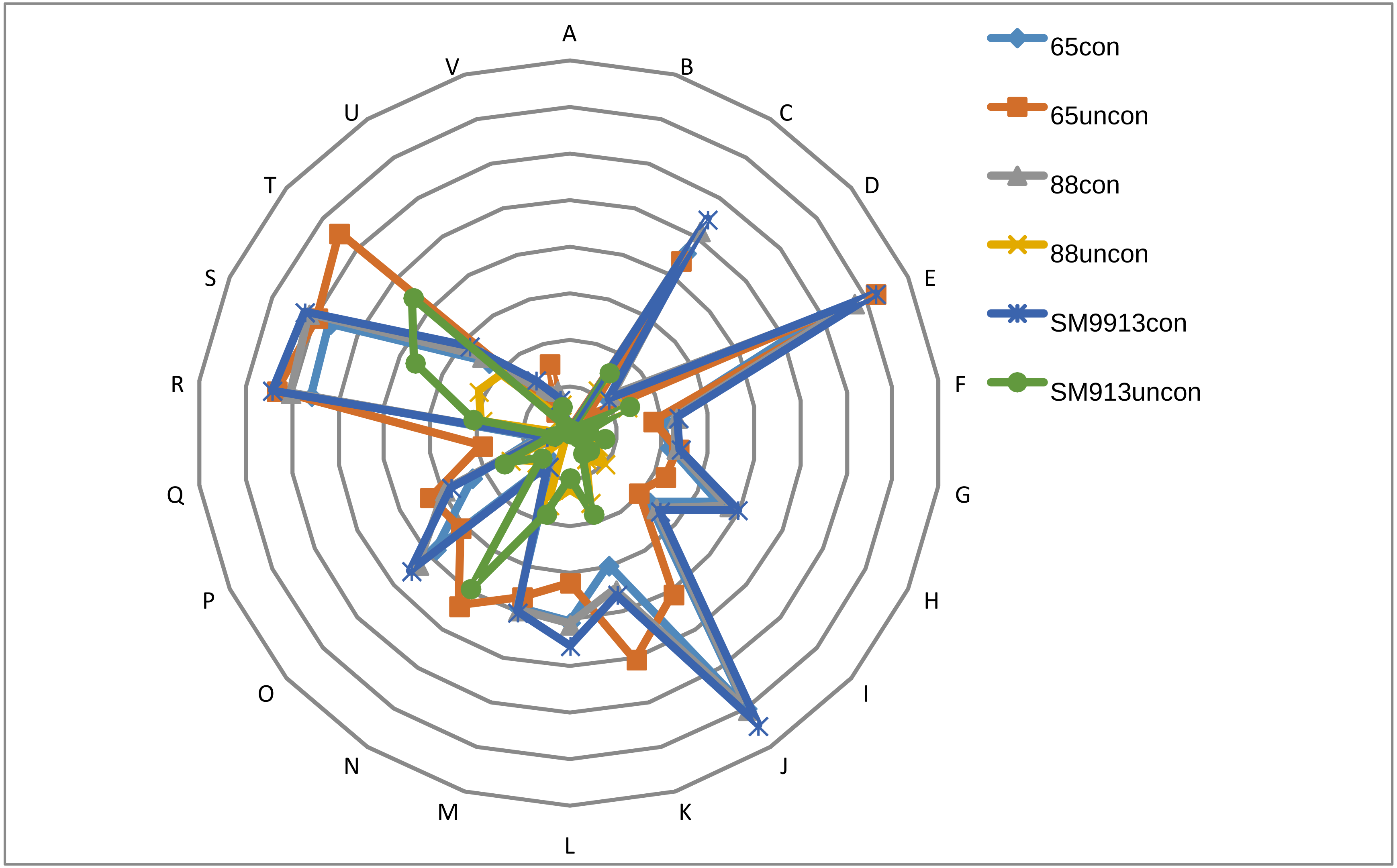
A
B
C
D
E
F
G
H
I
J
K
L
M
N
O
P
Q
R
S
T
U
V
65con
65uncon
88con
88uncon
SM9913con
SM913uncon

Figure S3. COG categories of two *Pseudoalteromonas* isolates (T1lg65 and T1lg88) with opposite quorum sensing-related traits analyzed by using *Pseudoalteromonas* sp. SM9913 as control. Difference between T1lg65 and T1lg88 orthologous proteins pertaining to COG T&J was special. 'T' means Signal transduction mechanisms; 'J' means Translation, ribosomal structure and biogenesis. The number of non-conserved sequence of 'T' in T1lg65 was higher than that of non-conservative sequence in strain SM9913. The numbers of conserved sequences of 'J' in T1lg65, T1lg88 and SM9913 were high, and The numbers of non-conserved sequence of 'J' in T1lg65 was less. A: RNA processing and modification; B: Chromatin structure and dynamics; C: Energy production and conversion; D: Cell cycle control, cell division, chromosome partitioning; E: Amino acid transport and metabolism; F: Nucleotide transport and metabolism; I: Lipid transport and metabolism; J: Translation, ribosomal structure and biogenesis; K: Transcription; L: Replication, recombination and repair; M: Cell wall/membrane/envelope biogenesis; N: Cell motility; O: Posttranslational modification, protein turnover, chaperones; P: Inorganic ion transport and metabolism; Q: Secondary metabolites biosynthesis, transport and catabolism; R: General function prediction only; S: Function unknown; T: Signal transduction mechanisms; U: Intracellular trafficking, secretion, and vesicular transport; V: Defense mechanisms.

(A)

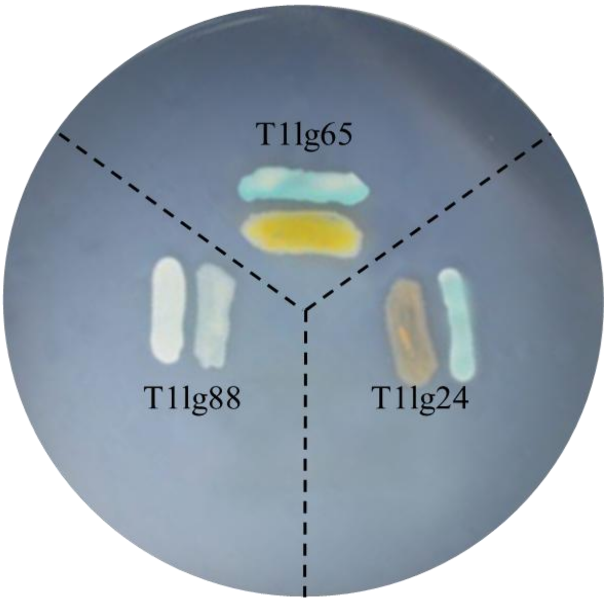
T1lg65
T1lg88
T1lg24

(B)

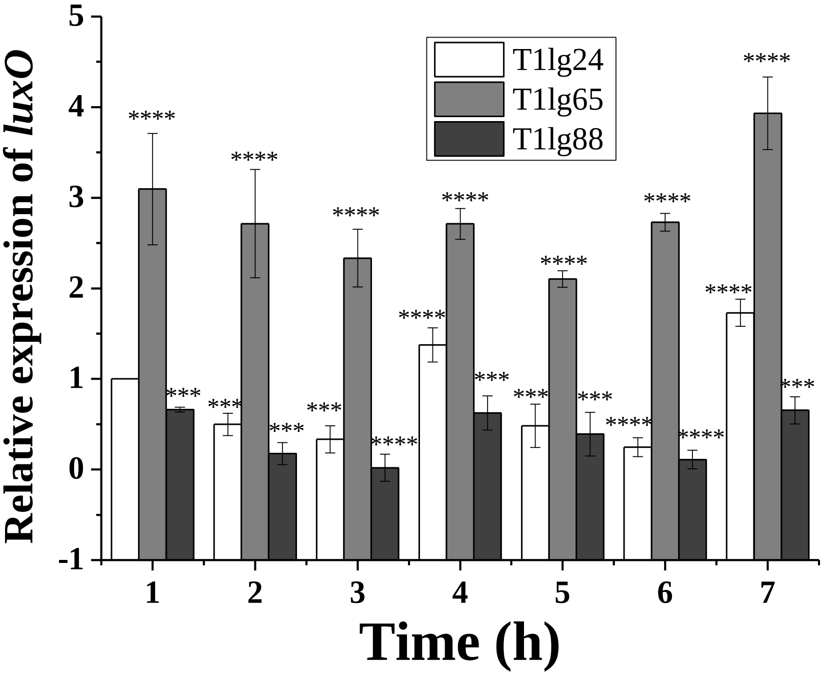
Relative expression of luxO
Time (h)
T1lg24
T1lg65
T1lg88
5
4
3
2
1
0
-1
1
2
3
4
5
6
7

(C)

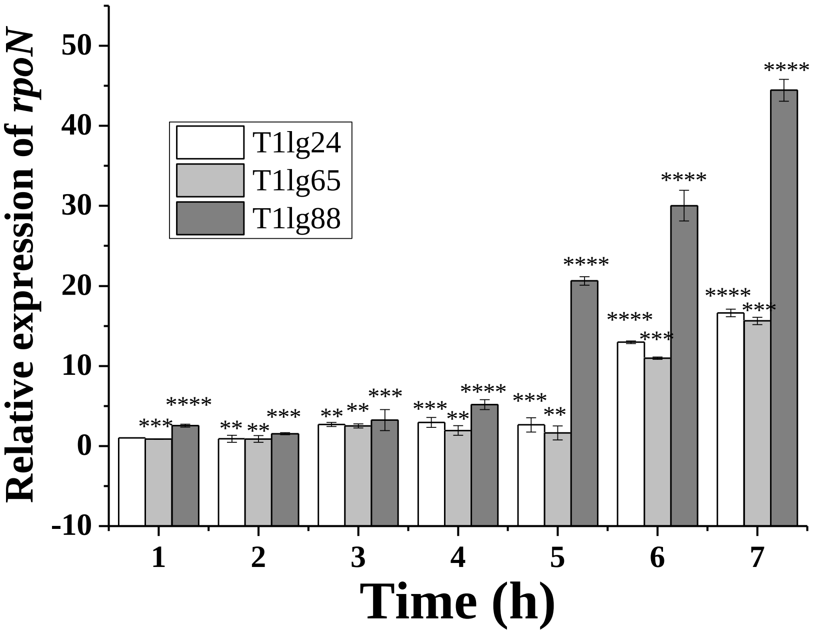

Figure S4. Quorum sensing-based colorization and relative expression of cognate regulatory genes in three selected *Pseudoalteromonas* isolates. (A) QS-based colorization of biosensor A136 using cross-feeding bioassay. T1lg65 seems to have the strongest QS-related traits, T1lg88 the weakest and T1lg24 moderate; (B) Relative expression of *luxO*, a positive regulator gene for QS; (C) Relative expression of *rpoN*, a negative regulator gene for QS. Triplicate reactions per experiment were performed for ANOVA test. Values that are not significantly different are not indicated. Statistical significance is respectively indicated by asterisks as follows: *, $p < 0.05$; **, $p < 0.01$; ***, $p < 0.001$; ****, $p < 0.0001$.

Supplementary S5: Biofilm formation assay using crystal violet

For each assay, 50 μL of glycerol cell stock, routinely stored at −80°C, was plated onto 2216E plate, and single colony was picked for inoculation in 5 mL of 2216E broth. Cultures were aerobically grown overnight with shaking at 30°C. The culture was diluted to around 1×10^9 CFU/mL in fresh broth, and 1 mL was added to a sterile glass test tube. Tubes were incubated for 72 h at 30°C without shaking. For crystal violet staining, cultures were removed. Next, tubes were washed with water for three times and then dried at 60°C for 30 min. One milliliter of a 1% crystal violet solution was added, and the tubes were incubated on a rocker at room temperature for 30 min. Unbound crystal violet was washed off with water, and the tubes were dried at 37°C. Bound crystal violet was dissolved into 1 mL of 33% glacial acetic acid and then poured into cuvettes. Finally, absorbance at 570nm was measured.

Reference: Wang PX, Zeng ZS, Wang WQ, Wen ZL, Li J, Wang XX. 2017. Dissemination and loss of a biofilm-related genomic island in marine *Pseudoalteromonas* mediated by integrative and conjugative elements. Environmental Microbiology 19:4620-4637.

Swimming motility assays

For each assay, a 50-μl single-use glycerol stock, routinely stored at −80°C, was plated onto a 2216E plate, and a single colony was picked for inoculation in 5 mL of 2216E broth. Cultures

were grown aerobically with shaking overnight at 30°C. Next, cell cultures were standardized to an optical density at 600nm of around 0.6, and 0.5μL of sample was spotted onto the center of 2216E plates with 0.3% agar. After dry for 30 min at room temperature, the plates were incubated upright for 12 h at 30°C, and swimming zone diameters were quantified using vernier calliper.

Reference: Qin QL, Li Y, Zhang YJ, Zhou ZM, Zhang WX, Chen XL, Zhang XY, Zhou BC, Wang L, Zhang YZ. 2011. Comparative genomics reveals a deep-sea sediment-adapted life style of *Pseudoalteromonas* sp. SM9913. ISME J 5:274-284.

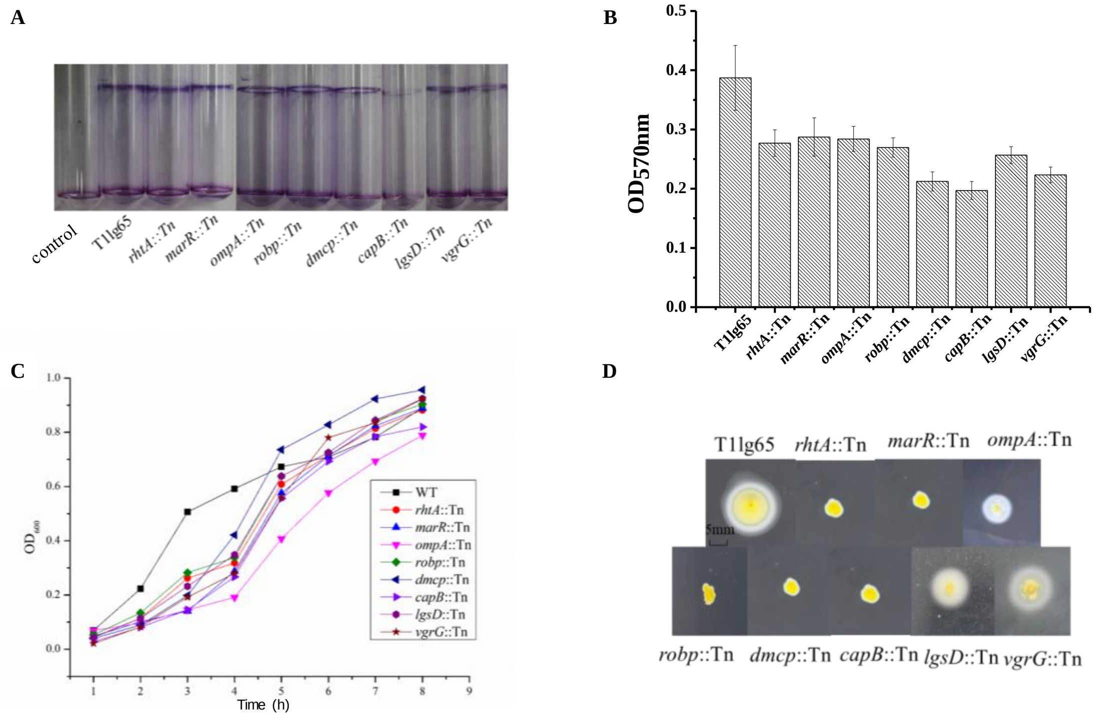

Figure S5. The consequence of gene mutation derived from Tn*10* disruption in this study. (A) Crystal violet staining was used to acquire biofilm formation efficacy in eight mutants; (B) and biofilm formation was evaluated based on detection of bound crystal violet at OD_{570nm}; (C) Growth status of eight mutants from strain T1lg65; (D) Swimming motility experiments for eight mutants. Control: *Shewanella oneidensis*.

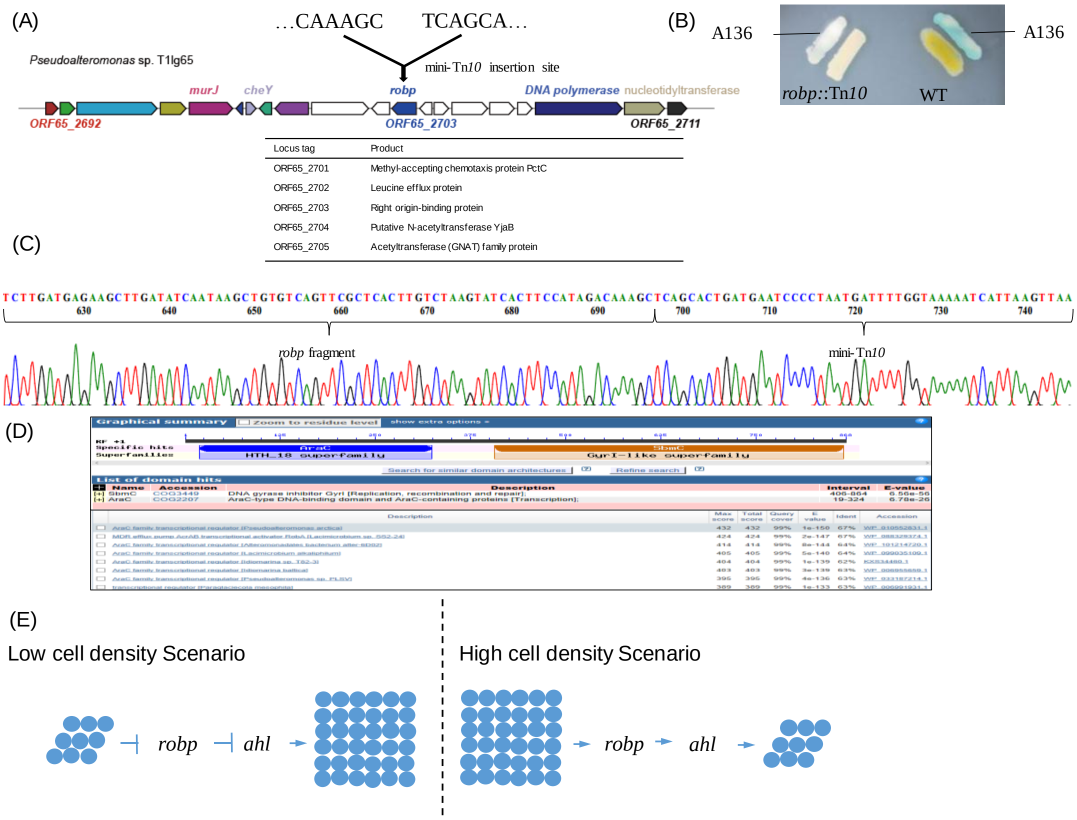

Locus tag	Product
ORF65_2701	Methyl-accepting chemotaxis protein PctC
ORF65_2702	Leucine efflux protein
ORF65_2703	Right origin-binding protein
ORF65_2704	Putative N-acetyltransferase YjaB
ORF65_2705	Acetyltransferase (GNAT) family protein

Figure S6. Characterization of *robp* essentiality for QS regulation by using Tn-Seq technology. (A) *robp* mutant achieved by using mini-Tn*10* mutagenesis; (B) Colorization difference of biosensor A136 between wild-type of isolate T1lg65 (WT) and mutant of T1lg65 with disrupted *robp* gene (*robp*::Tn*10*); (C) Sequencing identification of Tn*10*-inserted gene in mutant; (D) Functional annotation of conserved domains carried by *robp*; (E) Proposed working trade-offs of *robp*. Arrows and short lines indicate positive regulation (activation) and negative regulation (inhibition), respectively, under different cell densities. SbmC: proposed DNA gyrase inhibitor GyrI; AraC: AraC-type DNA-binding domain; *ahl*: acyl-homoserine lactone synthase gene (*lasI*).

(A)

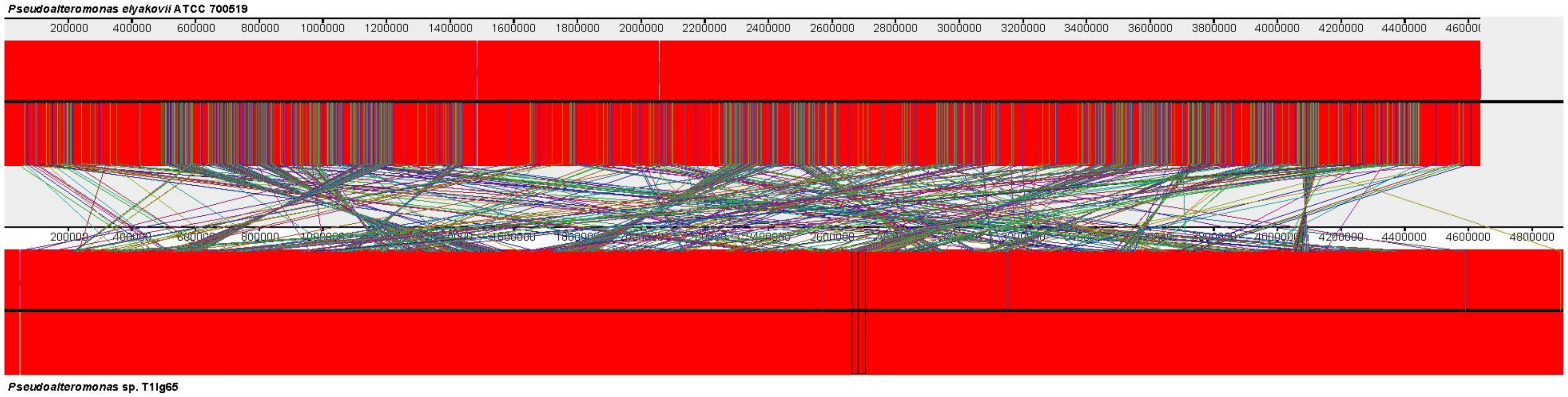
Pseudoalteromonas elyakovii ATCC 700519
200000 400000 600000 800000 1000000 1200000 1400000 1600000 1800000 2000000 2200000 2400000 2600000 2800000 3000000 3200000 3400000 3600000 3800000 4000000 4200000 4400000
4200000 4400000 4600000 4800000
Pseudoalteromonas sp. T1lg65

(B)

Robp

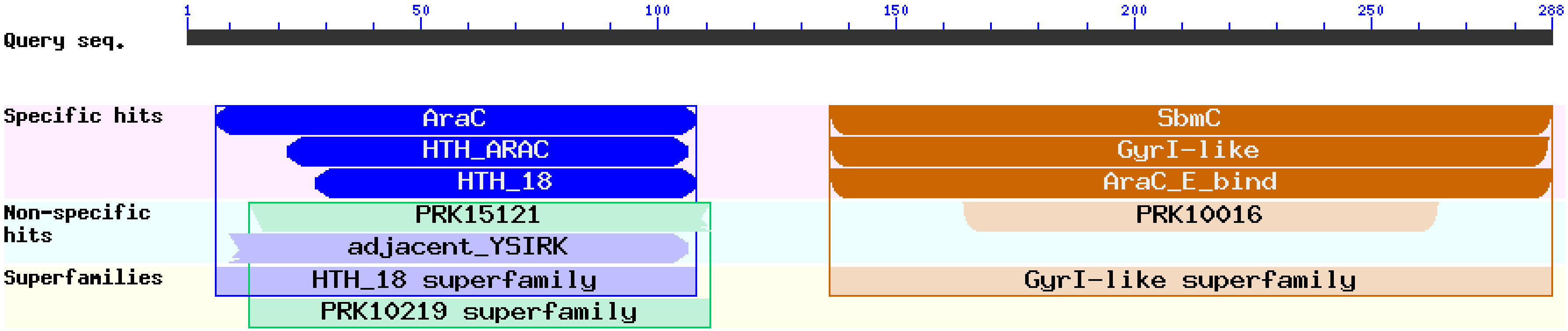
1
50
100
150
200
250
288
Query seq.
Specific hits
AraC
HTH_ARAC
HTH_18
SbmC
GyrI-like
AraC_E_bind
Non-specific hits
PRK15121
adjacent_YSIRK
PRK10016
Superfamilies
HTH_18 superfamily
PRK10219 superfamily
GyrI-like superfamily

(C)

VqsM

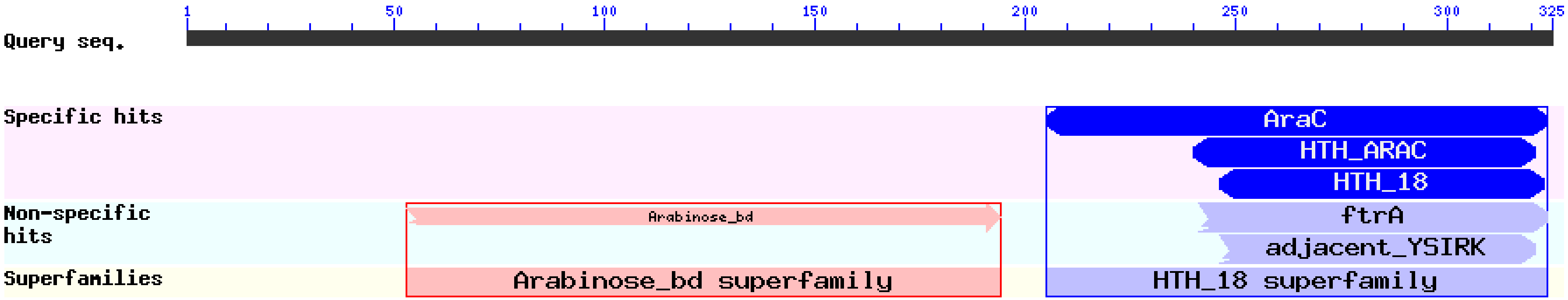

Figure S7. Sequence and phylogenetic analysis of Robp. (A) Co-linear alignment for T1lg65 and *Pseudoalteromonas elyakovii*, and no Robp homolog was predicted in phylogenetically-related *P. elyakovii* strain ATCC 700519; (B) Conserved domains of Robp in strain T1lg65. Of note, the black box indicates the '*robp*' island; (C) Conserved domains of VqsM in *P. aeruginosa* PAO1 (Liang, H. *et al*. Nucleic Acids Research 2014, 42, 10307-10320). VqsM is Robp homolog. GyrI: GyrI-like small molecule binding domain; AraC: AraC-type DNA-binding domain and AraC-containing proteins; Arabinose_bd: Arabinose-binding domain of AraC transcription regulator; HTH: Helix-turn-helix domain.
